# Z‐Scheme Photocatalytic Systems for Solar Water Splitting

**DOI:** 10.1002/advs.201903171

**Published:** 2020-02-13

**Authors:** Boon‐Junn Ng, Lutfi Kurnianditia Putri, Xin Ying Kong, Yee Wen Teh, Pooria Pasbakhsh, Siang‐Piao Chai

**Affiliations:** ^1^ Multidisciplinary Platform of Advanced Engineering Chemical Engineering Discipline School of Engineering Monash University Jalan Lagoon Selatan 47500 Bandar Sunway Selangor Malaysia; ^2^ Mechanical Engineering Discipline School of Engineering Monash University Jalan Lagoon Selatan 47500 Bandar Sunway Selangor Malaysia

**Keywords:** artificial photosynthesis, electron mediators, hydrogen, water splitting, Z‐scheme

## Abstract

As the world decides on the next giant step for the renewable energy revolution, scientists have begun to reinforce their headlong dives into the exploitation of solar energy. Hitherto, numerous attempts are made to imitate the natural photosynthesis of plants by converting solar energy into chemical fuels which resembles the “Z‐scheme” process. A recreation of this system is witnessed in artificial Z‐scheme photocatalytic water splitting to generate hydrogen (H_2_). This work outlines the recent significant implication of the Z‐scheme system in photocatalytic water splitting, particularly in the role of electron mediator and the key factors that improve the photocatalytic performance. The Review begins with the fundamental rationales in Z‐scheme water splitting, followed by a survey on the development roadmap of three different generations of Z‐scheme system: 1) PS‐A/D‐PS (first generation), 2) PS‐C‐PS (second generation), and 3) PS‐PS (third generation). Focus is also placed on the scaling up of the “leaf‐to‐tree” challenge of Z‐scheme water splitting system, which is also known as Z‐scheme photocatalyst sheet. A detailed investigation of the Z‐scheme system for achieving H_2_ evolution from past to present accompanied with in‐depth discussion on the key challenges in the area of Z‐scheme photocatalytic water splitting are provided.

## Graphical abstract

The significant implication of Z‐scheme systems of different generations in photocatalytic water splitting, from past to present, is outlined and reviewed. The rationales and mechanisms in Z‐scheme systems, followed by a survey on the development roadmap of Z‐scheme systems from the first to third generation are presented. Lastly, the rapid emergence of particulate Z‐scheme photocatalyst sheets is outlined.

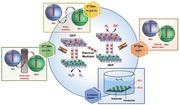

## 1 Introduction

### 1.1 Research Background

Owing to the global energy consumption continues to escalate and the incapability of energy replenishment from finite sources of fossil fuels to fulfill such needs, a diffusion of energy to more promising renewable carriers is anticipated in the near future. Presently, renewable energy shares an ≈3.6% of the global primary energy mix with a projected rising trend.^1^ Particularly, solar energy displayed the highest annual capacity increment of 31.3%, which is accounted to 99 GW from 2015 to 2016.^2^ The highly abundant energy from the Sun (173 000 TW) is an unexploited resource, and in this regard, scientists have begun to take advantage of this inexhaustible energy source.^3^ The versatility of solar energy conversion into different forms of energy, for instance, electricity from solar photovoltaic (PV) and heat from concentrating solar thermal power (CSP) have gained incessant attention worldwide. On the other hand, solar energy can also be converted and stored in chemical form, i.e., solar fuels (hydrocarbons and hydrogen, H_2_) by artificial photosynthesis mean. In this context, solar fuels bestow the advantages of sustainable mobility as compared to direct solar‐to‐electricity approach, attributed to the storage of solar energy in a medium carrier. Thus, solar‐to‐chemical conversion confers an intriguing route to harvest energy from the Sun to solve the on‐going severe energy deficit.^4^

**Figure 1:**
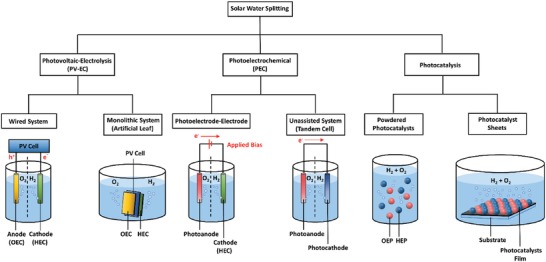
Schematic illustration of solar water splitting technologies: PV‐EC, PEC, and photocatalysis.

Among the solar fuels, H_2_ is worth noting as a potential candidate to become one of the future front runners in the energy mix ascribed to its nonpolluting nature and high energy density.^5^ A prevenient work on photocatalytic splitting of water using TiO_2_ photoelectrodes by Fujishima and Honda in 1972 marked the dawn of new era in solar H_2_ harvesting using artificial photosynthesis.^6^ Photocatalytic overall water splitting is a light‐driven energetically uphill reaction that demands a potential of 1.23 eV per electron to convert water molecules (H_2_O) into oxygen (O_2_) and the desirable H_2_, as shown in Equation (1).^7^ According to Equations (2) and (3), two electrons and four holes are required for H_2_ evolution reaction (HER) and its O_2_ evolution reaction (OER) counterpart, respectively. To date, several techniques for solar H_2_ production are being explored including photovoltaic‐electrolysis (PV‐EC), photoelectrochemical (PEC) and photocatalysis, as delineated in **Figure** **1**. Typically, PV‐EC utilizes a pair of electrolyzers, i.e., O_2_ evolution catalyst (OEC) as anode and H_2_ evolution catalyst (HEC) as cathode to split water via the independent power generated from solar PV (light absorber). In a conventional wired PV‐EC system, the PV cell and electrolyzers are constructed independently where solar PV is free from water to eliminate the concern of stability against corrosion in aqueous medium. Thus, the configuration of the PV unit and electrolyzers can be freely modulated. However, the disadvantage of wired PV‐EC system is the complexity of the design. As of recent, the development of monolithic system of PV‐EC by compact assembly of PV light absorber and electrolyzers confers an intriguing approach to construct wireless device (artificial leaf) with less complexity and omit the need of an external connection.^8^ Even so, the artificial leaf often delivers lower efficiency as compared to wired system due to the less efficient charge transfer via Ohmic contact

(1) $$\text{Overall}\;\text{water}\;\text{splitting}:2{\text{H}}_{2}\text{O}\;\to \;2H_{2}+\;{\text{O}}_{2}\;\left(\Delta G^{\text{o}}=237.2\;\text{kJ}\;{\text{mol}}^{-1}\right)$$

(2) $$\text{HER}:2{\text{H}}^{+}+\;2{\text{e}}^{-}\to \;{\text{H}}_{2}$$

(3) $$\text{OER}:\;2{\text{H}}_{2}\text{O}\;+\;4{\text{h}}^{+}\to {\text{O}}_{2}+\;4{\text{H}}^{+}$$

In view of PEC, the system is generally constructed in the photoelectrode–electrode configuration: 1) n‐type photoanode‐HEC cathode or 2) p‐type photocathode‐OEC anode. Different from PV‐EC, PEC photoelectrode is responsible for both light absorption and redox reaction. However, PEC setup with single light absorber requires external bias voltage to offset the overvoltages and other losses. An unassisted PEC water splitting reaction can be realized in a tandem cell which comprises of a photoanode for OER and a photocathode for HER. As compared to PV‐EC and PEC, particulate photocatalysis shows a more appealing route to generate H_2_ due to its simplicity and low cost since the system only requires light and water. Complicated setup and the application of external applied bias can be exempted which in turn offers an energy‐efficient strategy to generate H_2_ fuel. From past to present, particulate photocatalysis is conducted in powder suspension form. As of recent, the development of particulate photocatalyst sheets with localized photocatalyst powder on a substrate demonstrates an appealing strategy for potential scalability of photocatalysis.^9^ While this semiconductor‐mediated photocatalysis is potentially useful, the cost for renewable H_2_ production is outweighed by their efficiency. However, practical mass production might be realized in the future if revolutionary research studies on photocatalytic H_2_ production continue to grow.

### 1.2 Rationales and Mechanisms of Photocatalytic Water Splitting

Photocatalysts are generally made from semiconductors. Unlike the continuum electronic states of metal, the unique electronic structure of semiconductor which consists of an energy void region extended from the top of filled valence band (VB) to the bottom of vacant conduction band (CB) enables the promotion of ground state electron in VB to higher energy CB upon light irradiation. This phenomena, which is also known as bandgap photoexcitation, only occurs when the photocatalyst is induced by a photon absorption with energy equal or greater than the energy difference between the two energy levels or bandgap energy (*E*_g_).^10^ As a result, photoexcited electron (e^−^) will be accommodated in higher energy state CB, in turn leaving an empty hole (h^+^) in the lower energy state VB. After the initial photoexcitation process, there is a lifetime in nanosecond regime for the photoinduced electrons and holes to migrate to the surface of photocatalysts and eventually participate in oxidation–reduction reaction with the adsorbed reactants. However, electron–hole pairs recombination is in competition with the charge transfer process which impedes the supply of photogenerated electrons and holes.^11^ This is ascribed to the flash recombination time of photogenerated charge carriers which is in the order of 10^−9^ s. Since the time for chemical reaction of photocatalyst with the surface adsorbed molecules is in the range of 10^−8^ to 10^−3^ s, de‐excitation is much slower than excitation process which makes the recombination of charge inevitable.^12^

**Figure 2:**
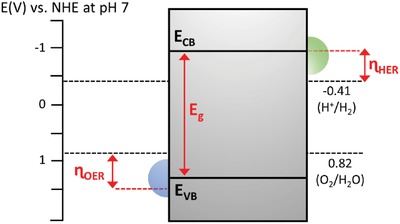
Fundamental photocatalytic water splitting mechanism in particulate system. The potential difference between CB and H^+^/H_2_ is abbreviated as *ɳ*_HER_ while the potential difference between VB and O_2_/H_2_O is abbreviated as *ɳ*_OER_.

Furthermore, thermodynamic law places a constraint for the redox reaction on the surface of photocatalysts. The law indicates that for a reduction reaction to occur, the potential level of CB must be more negative or analogously higher than the redox potential of lowest unoccupied molecular orbital (LUMO) of the acceptor molecule. Whereas for an oxidation reaction to proceed, the VB potential must be more positive or analogously lower than the redox potential of highest occupied molecular orbital (HOMO) of the donor molecule. This governs the tendency of acceptor or donor molecules to be reduced or oxidized which directly imply the probability of H_2_ and O_2_ production. Thus, for efficient H_2_ and O_2_ evolution, CB potential of the photocatalyst must be more negative than H_2_ production level, H^+^/H_2_ (−0.41 V vs normal hydrogen electrode (NHE) at pH 7. at pH 7) while VB potential has to be more positive than water oxidation level, O_2_/H_2_O (+0.82 V vs NHE at pH 7).^7^ The fundamental photocatalytic water splitting mechanism in particulate system is delineated in **Figure** **2**.

In this context, semiconductor plays a significant role in three major process steps of the heterogeneous photocatalysis: 1) bandgap photoexcitation to induce formation of electron–hole pairs, 2) migration of delocalized charge carriers to the surface of photocatalysts, and 3) provide active sites for the subsequent oxidation and reduction process.^13^ Thus, the photocatalytic performance is highly influenced by the electronic structure, bulk structure and surface structure of the photocatalysts. In other words, photocatalysts are desirable to possess narrow bandgap and strong redox ability. The former implies that a small bandgap could extend the photoresponsiveness of the photocatalysts for broader range of solar light utilization. Contrastingly, the latter suggests that a higher CB potential and a lower VB potential are more thermodynamically favorable for the respective reduction and oxidation reaction. However, more negative CB and positive VB will widen the bandgap of the photocatalysts which eventually resulted in the poor responsiveness of light. Both aspects of small bandgap and large overpotential are mutually significant but at the same time, they are exclusive.

### 1.3 Photocatalytic Systems for Water Splitting

**Figure 3:**
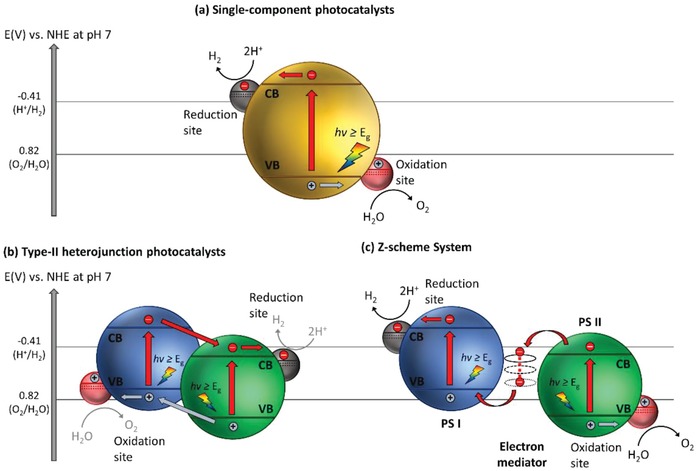
Schematic illustration of a) single component photocatalysts, b) type‐II heterojunction photocatalysts, and c) Z‐scheme photocatalytic system.

To date, there are two primary approaches for photocatalytic overall water splitting. First approach is to split water using single‐component photocatalysts through one‐step photoexcitation. Based on the aforementioned rationales, it is desirable to have a narrow bandgap photocatalyst to ease the photoactivation but at the same time retaining a considerable large overpotential level. This leads to better reduction and oxidation of the reactants owing to a more negative level of CB potential and a more positive level of VB potential are being applied thermodynamically. Thus, single‐component photocatalysts should have sufficiently small bandgap to be activated by light and possess suitable band‐edge potentials to achieve overall water splitting. However, it is clear that the two aspects above are mutually exclusive. Generally, it is extremely difficult and challenging for single‐component photocatalysts to fulfill such stringent requirements and hence, this greatly limits the potential photocatalyst candidates for one‐step water splitting. Thus, it is not feasible for single‐component photocatalysts to simultaneously dominate the properties of small bandgap and strong redox ability. Besides, single‐component photocatalysts also suffer from rapid recombination of photogenerated electron–hole pairs.^14^ Schematic illustration of charge transfer mechanism of single‐component photocatalysts in water splitting is demonstrated in **Figure** **3**a.

Owing to the shortcoming in single‐component photocatalytic system, extensive research efforts have been devoted over the years in tailoring the configuration of photocatalysts, for instance, forming type‐II heterojunction nanocomposites using two semiconductors in order to improve their charge separation efficiency and enhance the photocatalytic performance. Designing proper heterojunction‐type photocatalytic system can induce the isolation of electrons and holes in two separated locations, which in turn bolstering the lifetime of photogenerated carriers.^15^ Gas evolution occurs in two separated photocatalysts, i.e., HER on H_2_ evolution photocatalysts (HEP) and OER on O_2_ evolution photocatalysts (OEP). Despite the competency of heterojunction‐type semiconductor nanocomposites in facilitating charge separation, redox ability of the photocatalytic system is weakened due to the migration of electrons and holes to more electropositive CB and electronegative VB potential ascribed to the nature of charge transfer, as depicted in Figure 3b.

To overcome these bottlenecks, the second approach of photocatalytic system introduces an anisotropic configuration of two photocatalysts and an electron mediator known as the Z‐scheme photocatalytic system to perform water splitting through two‐step photoexcitation process. Inspired by natural photosynthesis in green plants, biomimetic artificial Z‐scheme photocatalytic system can simultaneously exhibit the following three requirements, which are lacking from both single‐component photocatalysts and heterojunction‐type nanocomposites: 1) small bandgap, 2) suitable band‐edge position with considerable large overpotential, and 3) suppression in electron–hole pairs recombination.^16^ Artificial Z‐scheme photocatalysis or known as the two‐step photocatalytic system achieves efficient water splitting through the synergistic actions of two isolated photocatalysts in which one of them serves as the reduction site (photosystem I or PS I) while the other contributes as the oxidation site (photosystem II or PS II). An electron transport chain known as the mediator is used to facilitate the electron flow between the two photosystems. Different from heterojunction‐type semiconductor composites, Z‐scheme enables a unique profile of electron flow attributed to the incorporation of an electron‐relaying channel. Upon photoexcitation, electrons from VB of both PS I and II will be excited to CB, leaving the photogenerated holes in the VB. The photogenerated electrons in PS II will be subsequently transported by the electron mediator to recombine with the holes from VB of PS I via Ohmic contact.^17^ This peculiar type of vectorial electron transfer allows electrons and holes to be accommodated in two separated photocatalysts at the same time retaining strong redox abilities. Thus, PS II is hole‐rich photocatalysts for OER while PS I is accumulated with electrons for HER. Hence, a lower change in Gibbs free energy is needed for a Z‐scheme system to drive each photosystem as compared to single‐component photocatalysts and heterojunction‐type composites.^7^ As displayed in Figure 3c, it can be clearly visualized that the rational design of Z‐scheme system bestows an efficient charge isolation in separated location with a relatively large overpotential which is sufficiently to govern the excellent redox reaction. A more detailed and comprehensive review on Z‐scheme photocatalytic system will be discussed henceforth.

## 2 Fundamental in Z‐Scheme Photocatalytic System

### 2.1 Historical Background and Development

Beginning with the pioneering discussion on two light reactions and two pigment systems by Rabinowitch on the statement of James Frank in 1945, numerous interests have peaked on mimicking the natural photosynthesis of green plants in harvesting solar energy.^18^ The ground‐breaking idea of Z‐scheme photocatalytic system model was firstly proposed by Bard in 1979 with the concept of employing an electron mediator as a charge transporting channel between two photosystems for the reduction and oxidation processes, respectively.^19^ This proposed system was inspired by the photosynthesis mechanism in plants, where photosystems I and II (PS I and II) can harvest photon energy up to 700 and 680 nm for CO_2_ reduction into carbohydrate and H_2_O oxidation into O_2_ with quantum yield of approximate unity.^16^ The very first key demonstration of Z‐scheme system in photocatalysis was introduced in 2001, where IO_3_^−^/I^−^ shuttle redox couple (electron acceptor/donor pair or known as A/D pair) was implemented as an ionic mediator to govern the charge transfer between two semiconductors as photosystems.^20^ This marks the beginning of first generation Z‐scheme system (PS‐A/D‐PS) with ionic redox pairs that work in reversible way under liquid phase. However, redox pair‐aided Z‐scheme system often suffers from inevitable drawbacks, for instance, backward reaction due to reversibility of ionic mediator which will impose a strong competition for forward redox reaction ascribed to the back donation of carriers.^21^ Besides, the redox pairs will absorb light to a certain extent which greatly reduce the available photons for forward reaction, known as shielding effect.^22^

With that in mind, an all‐solid‐state Z‐scheme system with Au as electron mediator was then introduced in 2006 which opened a new horizon for low resistance electron transfer via Ohmic contact.^23^ In this context, ionic electron mediator was substituted by metal conductor, constructing a Z‐scheme configuration of PS I‐conductor‐PS II system. Unlike the inefficient redox shuttle, the ingenious arrangement of all‐solid‐state Z‐scheme system utilizing a conductor as the mediator (second generation) renders a more promising electron relaying ability. It provides an interparticle electron transfer which greatly trim the distance of electron flow from PS II to PS I. On top of that, with the absence of redox pair, backward reaction and shielding effect can be perfectly prevented.^24^ However, the use of rare and expensive noble metal as the conductor greatly limits the feasibility of this system for wide application. More recently, there is a huge interest in designing next generation all‐solid‐state Z‐scheme system using carbon nanotubes (CNTs), graphene and carbon quantum dots (CQDs) as electron mediators.[qv: 17a,25] These findings could potentially extend the mediator candidates to a broader area.

One step forward in Z‐scheme photocatalysis can be witnessed in a mediator‐free two‐step photoexcitation system. As reported by Wang et al. in 2009, Z‐schematic vectorial electron transfer profile was found in coupling of ZnO and CdS via chemical formation method.^26^ The solid–solid contact interface, namely PS‐PS system or direct Z‐scheme (third generation), can confer a potent platform to induce the formation of internal electric field for vectorial electron flow. Besides, the concept of PS‐PS system was extended by Sasaki et al. via assembling of SrTiO_3_:Rh (PS I) and BiVO_4_ (PS II) using pH adjustment.^27^ The fabrication of this PS‐PS system using interparticle electrostatic adsorption for connecting PS I and II is known as physical formation method.^24^ PS‐PS systems, whether formed by chemical or physical means, preserve the advantages of PS‐C‐PS system by semiconductors alone without the need of an external mediator. However, the formation of internal electric field at the solid–solid contact interface is strongly dependent on the nature of both semiconductors and thus, both PS‐C‐PS and PS‐PS systems have their own advantages.

**Figure 4:**
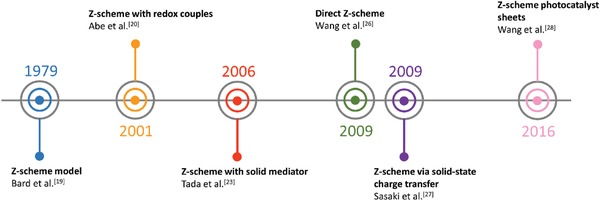
Timeline for the evolution in different generation of Z‐scheme.

More recently, scalable Z‐scheme photocatalyst sheets system was proposed by Wang et al. which confers potential scalability for the augmentation of solar water splitting.^28^ Different from the forebear of powder photocatalysis, the thin film form of Z‐scheme which comprises of dual‐layer particulate sheets with top layer to be photocatalysts embedded on an underlying electron mediator offers an avenue to further extend the concept of Z‐scheme system. The Z‐scheme photocatalyst sheets encompass vectorial electron transfer between PS I and II via Ohmic contact with the underneath conductor layer.^29^ The rational configuration of Z‐scheme photocatalyst sheets can suppress the effect of H^+^ and OH^−^ concentration overpotentials and pH gradient which in turn compensate for the Ohmic drop (IR drop) between PS I and II as a contrast to conventional powder system.^30^ Based on the roadmap of evolution of Z‐scheme photocatalytic system shown in **Figure** **4**, it can be deduced that extensive efforts have been made throughout the years in order to exploit the mechanism of photosynthesis in nature and numerous studies have been devoted to developing light‐harvesting system which resembles photosynthesis in order to fully utilize solar energy. The following sections will focus on the fundamental rationales and mechanism of different Z‐scheme systems, their development and application in water splitting as well as the strategies to improve the photocatalytic efficiency.

### 2.2 Principles and Mechanisms in Natural Photosynthesis

**Figure 5:**
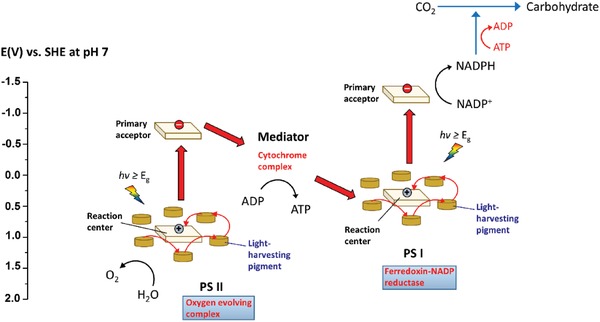
Graphical representation illustrating two‐step photoexcitation system in natural photosynthesis of green plant.

**Figure** **5** shows the two‐step photoexcitation mechanism of natural photosynthesis in green plants. As aforementioned, this photosynthesis system can convert CO_2_ and H_2_O into carbohydrate and O_2_ with quantum efficiency close to 100%. First, initial photoexcitation occurs when reaction center chlorophylls in PS I (λ ≤ 700 nm) and II (λ ≤ 680 nm) absorb photons from sunlight, leading to delocalization of charges to form electron–hole pairs.^31^ The photogenerated electrons in PS II will then be transported to quench the photogenerated holes in PS I via a shuttling channel, while the accumulated photons emancipated can simultaneously induce an electronic potential to convert adenosine diphosphate (ADP) to adenosine triphosphate (ATP).^32^ As a result, photogenerated electrons and holes will be isolated in PS I and II, performing reduction and oxidation processes at two separated locations. Oxidation of H_2_O into O_2_ happens in manganese calcium oxide cluster of PS II ascribed to the accumulated holes in HOMO of this unit, labelled as oxygen‐evolving complex. Whereas LUMO of PS I is full of electrons which in turn reduce nicotinamide adenine dinucleotide phosphate (NADP^+^) into NADPH, labelled as ferrodoxin‐NADP reductase unit.^24^ Working in conjunction with ATP, reduction power is provided to support the conversion of CO_2_ into carbohydrate.^33^ The electron cascade steps of vectorial charge flow profile in this two‐step photoexcitation system resembles the alphabet Z, which give rise to the name of Z‐scheme system. Its mechanism confers an efficient charge separation and demonstrates strong reduction and oxidation abilities within the system.

Inspired by natural photosynthesis, biomimetic artificial Z‐scheme photocatalytic system shows similar vectorial charge transport profile that features strong reducibility and oxidizability of PS I and II connected by an electron mediator. Besides, the nature of electron‐rich region in PS I can efficiently suppress photo‐degradation from self‐oxidation.^24^ Hence, the unique configuration of Z‐scheme can enhance the photostability of HEP as compared to single‐component HEP. Similarly, PS II is accumulated with photogenerated holes, which serves as a hole‐rich region, thus preventing PS II to be tormented by photoreduction. However, this statement implies that PS I should be a semiconductor with strong reduction ability, while PS II requires strong oxidizing ability to protect them from the corresponding reduction and oxidation reaction and, at the same time, promotes forward water splitting reaction. Besides, the number of photogenerated electron–hole pairs in Z‐scheme system is halved as compared to conventional photocatalytic system ascribed to the recombination of charge through the electron mediator. Even so, Z‐scheme system can efficiently surmount the bulk recombination and increase the lifetime of photogenerated charge carriers in the isolated photosystems.^34^

## 3 Current Status of Z‐Scheme Systems for Water Splitting

### 3.1 PS‐A/D‐PS System (First Generation)

#### 3.1.1 Mechanism of Electron Mediator

**Figure 6:**
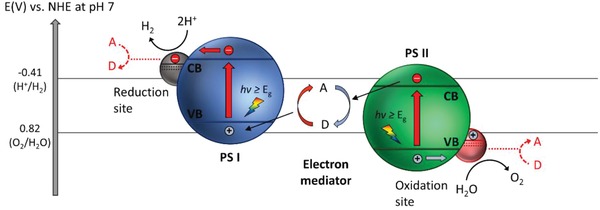
Schematic band energy diagram of PS‐A/D‐PS Z‐scheme system.

In an effort to imitate the natural photosynthesis, artificial Z‐scheme photocatalytic process can be firstly witnessed in redox‐mediated Z‐scheme by employing ionic mediator known as electron A/D pair.^35^ As delineated in **Figure** **6**, the redox‐mediated Z‐scheme, also named as PS‐A/D‐PS (first generation) system, consists of A/D pair as electron shuttle and two semiconductors as PS I and II. In such system, two sets of charge carriers will be delocalized in separated semiconductors, resulting in water splitting into its constituent parts of H_2_ and O_2_. The cascade type of vectorial electron transfer from PS II to PS I is governed by A/D pair with no physical contact between PS I and II. Initially, after generation of electron–hole pairs due to photoexcitation in both PS I and II, the electron acceptors will be reduced by the electrons from CB of PS II into electron donor, leaving photogenerated holes accumulated in VB of PS II (Equation (4)). Meanwhile, the produced electron donors will be then converted back into its oxidized form (electron acceptor) by the photogenerated holes from VB of PS I (Equation (6)). Consequently, photogenerated electrons from PS II are indirectly shuttled to recombine with photogenerated holes from PS I with the aids of A/D pair, resulting in efficient isolation of electrons and holes in PS I and II, respectively. Thus, the photogenerated charge carriers are accommodated in the highest possible CB and lowest VB of the system, offering a considerable large overpotential for the Z‐scheme reactions (Equations (5) and (7)).^21^ As such, this Z‐scheme system is feasible to drive photocatalytic overall water splitting with the continuous regeneration of redox pairs

(4) $$\text{A}\;+\;{\text{e}}^{-}\left(\text{CB}\;\text{of}\;\text{PS}\;\text{II}\right)\to \;\text{D}$$

(5) $$2{\text{H}}_{2}\text{O}\;+\;4{\text{h}}^{+}\left(\text{VB}\;\text{of}\;\text{PS}\;\text{II}\right)\;\to {\text{O}}_{2}+\;4{\text{H}}^{+}$$

(6) $$\text{D}\;+\;{\text{h}}^{+}\left(\text{VB}\;\text{of}\;\text{PS}\;\text{I}\right)\;\to \text{A}$$

(7) $$2{\text{H}}^{+}+\;2{\text{e}}^{-}\left(\text{CB}\;\text{of}\;\text{PS}\;\text{I}\right)\;\to {\text{H}}_{2}$$

However, electron A/D pair in PS‐A/D‐PS system can also react with the photogenerated electrons and holes in CB of PS I and VB of PS II, which cause inevitable decrease in effective number of charge carriers, as disclosed by the red dotted lines in Figure 6. Thus, it is rather difficult for a PS‐A/D‐PS Z‐scheme system to simultaneous evolve H_2_ and O_2_ gas in stoichiometric ratio ascribed to the backward reaction. With respect to this issue, surface treatments, for instance, metal co‐catalysts loading, deposition of rutile TiO_2_ and exchange of Cs‐H^+^ are some of the efforts to impede the backward reaction by preventing the adsorption of electron acceptors on PS I and electron donors on PS II.^36^ To date, the commonly employed redox mediators in PS‐A/D‐PS Z‐scheme system are IO_3_^−^/I^−^, Fe^3+^/Fe^2+^, [Co(bpy)_3_]^3+/2+^, [Co(phen)_3_]^3+/2+^, and VO_2_^+^/VO^2+^.^37^ The implication and development of PS‐A/D‐PS Z‐scheme system with different redox couples will be investigated and discussed henceforth.

#### 3.1.2 Simple Ion Redox Couples

##### Iodate/Iodine (IO_3_^−^/I^−^) Redox System

Early work of photocatalytic Z‐scheme system was demonstrated by Abe et al. using IO_3_^−^/I^−^ redox couple as an ionic electron mediator to interface two different types of TiO_2_, namely anatase and rutile as corresponding HEP and OEP.^20^ Aqueous iodine is competent as electron mediator ascribed to the wide range of its valences from −1 to +7. However, the valence charges of I^(0/1−)^ and I^(1−/5+)^ are more stable as redox cycles in aqueous medium as shown in Equations (8) and (9).^38^ The ionic pair of I_3_^−^/I^−^ is ordinarily implemented in dye‐sensitized solar cell, attributed to its facile reversible redox reaction and exhibits a low degree of photoabsorption which is only up to ≈500 nm.^39^ However, the application of Z‐scheme using I_3_^−^/I^−^ redox cycle is very limited due to the low reactivity of I_3_^−^ as electron acceptor and the restricted selection of photosystems.^40^ On the other hand, IO_3_^−^/I^−^ redox pair possesses standard potential that is close to I_3_^−^/I^−^ couple. Thus, the incorporation of IO_3_^−^/I^−^ as ionic redox mediator is feasible due to its electron nature

(8) $${\text{I}}_{3}{}^{-}+2{\text{e}}^{-}\;3{\text{I}}^{-}\;E^{\text{o}}\;\text{versus}\;\text{NHE}\;=\;+0.536\;\text{V}$$

(9) $${\text{IO}}_{3}{}^{-}+6{\text{e}}^{-}+\;3{\text{H}}_{2}\text{O}\to {\text{I}}^{-}+\;6{\text{OH}}^{-}\;E^{\text{o}}\;\text{versus}\;\text{NHE}\;=\;+0.670\;\text{V}$$

Even so, pH of the medium plays a significant role on the performance of IO_3_^−^/I^−^ redox cycle. This ionic pair exhibits larger contribution under increasing pH due to the shifting in standard potential according to Nernst equation.^34^ In the work by Abe the photocatalytic gas evolution of a NaI suspension containing Pt‐loaded anatase TiO_2_ (Pt‐TiO_2_‐A1) as PS I and rutile TiO_2_ (TiO_2_‐R2) as PS II was conducted under UV illumination.^33^ Sluggish gas evolution from the system can be clearly observed at pH 3 imputed to the dissociation into I_3_^−^ as the main redox product rather than IO_3_^−^, which is incompetent to serve as an efficient electron acceptor in photocatalysis as mentioned earlier. On the other hand, basic solution of pH 9 is the optimum condition for IO_3_^−^/I^−^ redox system to work efficiently ascribed to the presence of favorable IO_3_^−^/I^−^ ionic pairs rather than I_3_^−^/I^−^. The gradual increment in activities from pH 5 to 9 is believed to be the induction period of IO_3_^−^ production with certain amount of I_3_^−^, which leads to the nonstoichiometric evolution of H_2_ and O_2_. Besides, the accumulation of ineffective I_3_^−^ will also cause some shielding effect due to their large extent of light absorption. Thus, it can be concluded that IO_3_^−^/I^−^ redox cycle can function efficiently when pH is higher than 9 owing to the absence of by‐product of I_3_^−^, resulting in stoichiometric evolution of H_2_ and O_2_ with high activities. Besides, the rate of gas evolution in such a system is also strongly dependent on the ratio of IO_3_^−^ to I^−^. The higher concentration of I^−^ will cause competitive oxidation known as backward reaction. Hence, suitable pH and concentration of NaI are crucial to operate PS‐A/D‐PS system with IO_3_^−^/I^−^ redox mediator. The investigation of IO_3_^−^/I^−^ redox system with other classes of semiconductor will be discussed henceforth.

**Figure 7:**
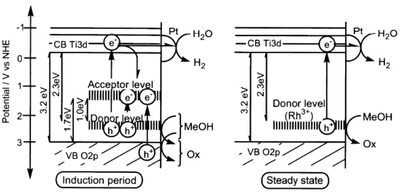
Effect of midgap states as electron donor level. Proposed band structure of Rh‐doped SrTiO_3_. Adapted with permission.^43^ Copyright 2004, American Chemical Society.

In 2002, Sayama et al. utilized Pt‐loaded SrTiO_3_ co‐doped with Cr and Ta (Pt‐SrTiO_3_:Cr, Ta) as HEP and Pt‐WO_3_ as OEP to govern IO_3_^−^/I^−^ Z‐scheme redox system.^41^ Despite the relatively large bandgap of pristine SrTiO_3_ (3.1 eV), many endeavors such as cation doping and loading of co‐catalysts have been devoted to enhancing the photoabsorption of this material in water splitting.^42^ The introduction of foreign noble metal ions (Rh, Mn, Ru, Ir, Cr, Ta, etc.) as dopants can induce new hybridized level above VB of SrTiO_3_ and tune the electronic band structure.^43^ These newly formed states can serve as an electron donor level, which indirectly reduce the bandgap of SrTiO_3_ (**Figure** **7**). However, the presence of such midgap states can also serve as a charge recombination center which decreases the photocatalytic performance. Hence the effects of cation doping into SrTiO_3_ cut both ways and an optimized doping degree is needed for improved activity. Attributed to the small ionic radius of Cr^3+^ and Ta^5+^, it can be speculated that Ti^4+^ ions in SrTiO_3_ lattice is substituted. In whole, the co‐doping of Cr and Ta metal ions can substantially booster the photocatalytic water splitting performance of SrTiO_3_ in IO_3_^−^/I^−^ redox system and improve the stability of the reaction.

**Figure 8:**
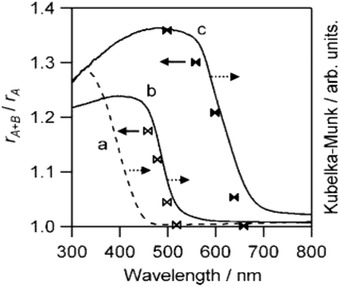
Degree of r_A+B_/r_A_ ratio with cutoff wavelength of light. UV–vis DRS of a) WO_3_, b) CaTaO_2_N, and c) BaTaO_2_N. Adapted with permission.^35^ Copyright 2009, American Chemical Society.

First demonstration of Z‐scheme water splitting with photoresponse up to 660 nm can be witnessed in IO_3_^−^/I^−^ redox system containing Pt‐BaTaO_2_N as HEP and Pt‐WO_3_ as OEP.^35^ Mixed tantalum oxynitrides have garnered considerable attention attributed to the strong hybridization between N 2p and O 2p orbitals which in turn offers a more electronegative VB and a smaller bandgap as compared to metal oxide semiconductors. The absorption edges of CaTaO_2_N and BaTaO_2_N are depicted in **Figure** **8**, which corresponds to 520 and 660 nm. Both Pt‐CaTaO_2_N and Pt‐BaTaO_2_N were able to perform H_2_ evolution under 5 × 10^−3^ m of NaI solution with production of IO_3_^−^ being detected. The amount of N_2_ generated was negligible during the reaction, which attested the absence of photocorrosion. More interestingly, a study employing ZrO_2_ as modifier on TaON can efficiently suppress the problem of photocorrosion, leading to exceptional AQY of more than 6% under monochromatic 420.5 nm in a system with Pt/WO_3_ as OEP and IO_3_^−^/I^−^ as redox pair.[qv: 36a]

##### Fe (Fe^3+^/Fe^2+^) Redox System

Similar to IO_3_^−^/I^−^ ionic couple, redox cycle of trivalent and divalent Fe ion (Fe^3+^/Fe^2+^) is another conventional A/D pair used in the early stage of PS‐A/D‐PS system.[qv: 37c] Interestingly, Fe^3+^/Fe^2+^ redox couple confers numerous advantages as compared to the ionic forebear of IO_3_^−^/I^−^ pair, attributed to the facile reduction process of Fe^3+^ to Fe^2+^. In this sense, Fe^3+^ is often being employed as an effective sacrificial reagent (electron acceptor) in photocatalytic water oxidation due to its one‐electron transfer nature.^44^ Thus, the incorporation of oxygen evolution co‐catalyst is not necessary for the efficient electron transfer from OEP, even though the presence of co‐catalysts can boost the performance. The redox cycle of Fe^3+^/Fe^2+^ ionic pair is shown in Equation (10), 38

(10) $${\text{Fe}}^{3+}+\;{\text{e}}^{-}\;{\text{Fe}}^{2+}\;E^{\text{o}}\;\text{versus}\;\text{NHE}\;=\;+0.771\;\text{V}$$

**Figure 9:**
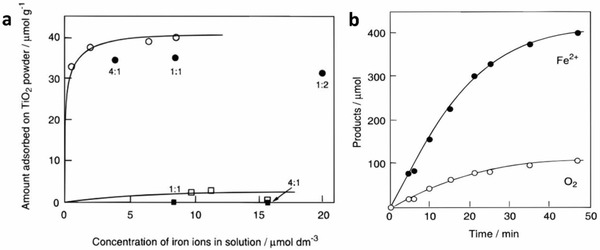
Properties of Fe^3+^/Fe^2+^ redox system. a) Adsorption isotherms of Fe^3+^ and Fe^2+^ on TiO_2_ powder. b) Photocatalytic generation of Fe^2+^ and O_2_ using Fe^3+^ as the electron acceptor. The reaction was carried out in 0.05 dm^3^ of aqueous FeCl_3_ containing TiO_2_ powder. Adapted with permission.^45^ Copyright 1997, American Chemical Society.

Besides, the unique adsorption properties of Fe^3+^/Fe^2+^ redox pair enable bolstering effect on the photocatalytic performance. As shown in **Figure** **9**a, the adsorption behaviors of both Fe^3+^ and Fe^2+^ on suspension containing rutile TiO_2_ were tested.^45^ It can be observed that Fe^3+^ is preferably adsorbed on TiO_2_ nanoparticles even with the presence of Fe^2+^ ions. Thus, Fe^3+^ will be reduced into Fe^2+^ by the photoexcited electrons from TiO_2_, while the photogenerated holes preferably oxidize water rather than Fe^2+^. This can be validated by the concurrent evolution of O_2_ gas and detection of Fe^2+^ from photocatalytic reaction of TiO_2_ under aqueous Fe^3+^ as shown in Figure 9b. However, such behavior of Fe^3+^/Fe^2+^ ionic pair only applicable to certain materials.^46^ Despite possessing the upper hand as compared to IO_3_^−^/I^−^ ionic pair, Fe^3+^/Fe^2+^ redox couples are only chemically stable in environment with pH less than 2.5. Precipitation of Fe(OH)_3_ from Fe^3+^ occurs under higher pH due to hydrolysis which constraint the application of this redox mediator. Besides, aqueous Fe^3+^ exhibits strong light absorption up to ≈450 nm which poses shielding effect to the system. Though FeCl_2_ only displays absorption of wavelength less than 250 nm, the photochemical oxidation of Fe^2+^ to Fe^3+^ might happen under UV irradiation.^34^ Nevertheless, careful attention should be paid to devise a Fe^3+^/Fe^2+^ redox system in order to exploit its function.

**Figure 10:**
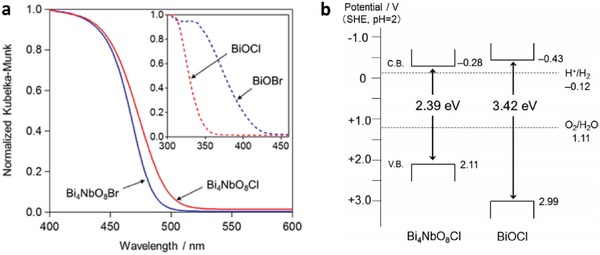
Optical properties of Sillén‐Aurivillius class of bismuth oxyhalides. a) UV–vis of Bi_4_NbO_8_Cl, Bi_4_NbO_8_Br, BiOCl and BiOBr. b) Schematic of band structure for Bi_4_NbO_8_Cl and BiOCl. Adapted with permission.^49^ Copyright 2016, American Chemical Society.

Early work of Fe^3+^/Fe^2+^ redox system can be witnessed in the incorporation of BiVO_4_ as PS II with Ru/SrTiO_3_:Rh as PS I.[qv: 37c] BiVO_4_ is one of the well‐documented OEPs in photocatalytic water splitting attributed to its strong hybridization between Bi 6s and O 2p orbitals, which leads to a more narrow bandgap (2.4 eV) as compared to WO_3_ (2.8 eV).^47^ With the employment of FeCl_3_ as mediator, BiVO_4_ can readily oxidize water into O_2_ and reduce Fe^3+^ into Fe^2+^ for the corresponding redox cycle to uptake holes from Ru/SrTiO_3_:Rh. As a result, the photocatalytic overall water splitting of such a system demonstrated an AQY of 0.3%, along with a long term stability up to 70 h.[qv: 37c] Owing to the facile reduction of Fe^3+^, the employment of co‐catalyst on OEP is not necessary. Recently, innovative research focuses on constructing Sillén‐Aurivillius class of bismuth oxyhalides, Bi_4_MO_8_X (M = Nb, Ta; X = Cl, Br) as efficient OEP. This class of material comprised of alternative stacking of halogen anionic blocks in between (Bi_2_O_2_)^2+^ layers.^44^ Taking Bi_4_NbO_8_Cl as the center of discussion, the strong orbital hybridization of Bi 6s with O 2p rather than Cl 3p bestows a more electronegative VB as compared to conventional bismuth oxyhalides.^48^ In other words, Sillén‐Aurivillius Bi_4_NbO_8_Cl possesses bandgap that is much smaller than its forebear BiOCl. As shown in **Figure** **10**, Bi_4_NbO_8_Cl and Bi_4_NbO_8_Br confer more redshifted light absorption compared to BiOCl and BiOBr, which offers a more favorable band structure for water oxidation. Consequently, visible‐light‐driven overall water splitting of Fe^3+^/Fe^2+^ redox system consisting of Bi_4_NbO_8_Cl and Ru/SrTiO_3_:Rh shows stoichiometric evolution of H_2_ (10.4 µmol h^−1^) and O_2_ gas (5.2 µmol h^−1^), respectively.^49^

**Figure 11:**
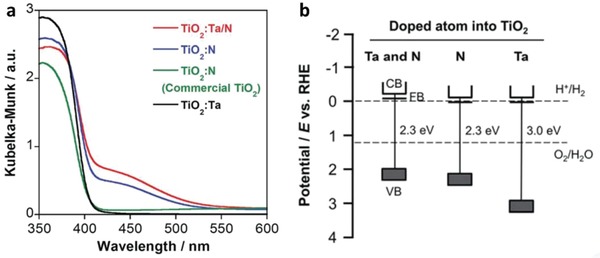
Optical properties of doped TiO_2_. a) UV–vis DRS of TiO_2_ and doped TiO_2_. b) Schematic illustration of band structure for TiO_2_ doped with different atoms. Adapted with permission.^52^ Copyright 2017, Royal Society of Chemistry.

TiO_2_ has been regarded as one of the most established semiconductors for photocatalytic application owing to its stability and suitable VB for water oxidation.^50^ However, the extend of function for TiO_2_ is greatly limited by its wide bandgap in various polymorphs, i.e., rutile (3.0 eV) and anatase (3.2 eV).^51^ In a recent work by Nakada et al., Ta and N atoms have been successful doped interstitially into the framework of rutile TiO_2_, forming titania with a narrow bandgap of 2.3 eV.^52^ As depicted in **Figure** **11**a, the intrusion of foreign atoms will induce an absorption tail in UV–vis under the visible region ascribed to the band trailing effect. The enhancement in light absorption is further validated by the shifting of VB after Ta and N co‐doping, leading to a smaller bandgap of TiO_2_ (Figure 11b). Consequently, RuO_2_‐loaded TiO_2_:Ta,N (OEP) and Ru/SrTiO_3_ (HEP) work well under both Fe^3+^/Fe^2+^ and IO_3_^−^/I^−^ redox systems, resulting in stoichiometric evolution of H_2_ and O_2_. Thus, this finding marks the endeavors in enhancing the function of TiO_2_ in Z‐scheme water spitting under visible light.

#### 3.1.3 Metal Complex Redox Couples

Other than the conventional simple ion redox couples, transition metal complexes such as cobalt‐based mediator [Co(bpy)_3_]^3+/2+^ and [Co(phen)_3_]^3+/2+^ show promising potential in driving PS‐A/D‐PS system attributed to their tunable standard potential and facile reversibility over a wide range of pH.^53^ However, the choice of HEP is crucial in order to operate under [Co(bpy)_3_]^3+/2+^ and [Co(phen)_3_]^3+/2+^ redox system due to their capability of generating Co^2+^ ions. A series of [Co(bpy)_3_]^3+/2+^ and [Co(phen)_3_]^3+/2+^ aqueous systems containing Pt/TiO_2_‐anatase, Pt/SrTiO_3_, Pt/SnNb_2_O_6_ and Pt/SrTiO_3_:Rh as HEPs were investigated by Sasaki et al. for their corresponding H_2_ evolution, as depicted in **Table** **1**.^54^ SrTiO_3_:Rh loaded with either Pt or Ru as co‐catalysts is the sole photocatalyst from the list that can produce H_2_ under aqueous solution of [Co(bpy)_3_]^3+/2+^ and [Co(phen)_3_]^3+/2+^. These findings elucidate the incompetency of TiO_2_ and undoped SrTiO_3_ in driving the oxidation of Co^2+^ ions into Co^3+^, resulting in the negligible photocatalytic gas evolution. Besides, it can be entrenched that the interstitial doping of Rh plays an important role in facilitating the oxidation of Co^2+^ according to Equation (11). In addition, the selection of materials for OEP is also crucial in order to accomplish efficient [Co(bpy)_3_]^3+/2+^ and [Co(phen)_3_]^3+/2+^ redox cycles. As shown in **Table** **2**, TiO_2_:Cr,Sb and BiVO_4_ demonstrate stoichiometric evolution of H_2_ and O_2_ gas, whereas WO_3_ renders much sluggish O_2_ rate which is well below stoichiometric value of 1:2 to H_2_. This phenomenon is well explained by the stronger oxidation potential of O 2p (WO_3_) orbital as compared to Bi 6s (BiVO_4_) and Cr 2p (TiO_2_:Cr,Sb) which induce the decomposition of Co‐complex from Co^3+^ rather than being oxidized into its redox counterpart Co^2+^. Thus, the more electropositive VB potential of WO_3_ reflects the shortcoming of cobalt‐based mediator ascribed to its insufficient stability under strong oxidation

(11) $${\text{Co}}^{3+}-\text{complex}+\;{\text{e}}^{-}{}_{\text{CB}}\left({\text{SrTiO}}_{3}:\text{Rh}\right)\to \;{\text{Co}}^{2+}-\;\text{complex}$$

**Table 1:** H_2_ evolution from aqueous solutions of [Co(bpy)_3_]^2+^, [Co(phen)_3_]^2+^ and Co^2+^ ions on various HEPs. Adapted with permission.^54^ Copyright 2013, American Chemical Society

Entry^a)^	Photocatalysts	Electron donor	Incident light [nm]	Initial activity [µmol h^−1^]
1	Pt(0.3 wt%)/TiO_2_‐anatase	[Co(bpy)_3_]^2+^	>300	0
2	Pt(0.3 wt%)/TiO_2_‐anatase	[Co(phen)_3_]^2+^	>300	0.2
3	Pt(0.3 wt%)/SrTiO_3_	[Co(bpy)_3_]^2+^	>300	0.02
4	Pt(0.3 wt%)/SrTiO_3_	[Co(phen)_3_]^2+^	>300	0
5	Pt(0.3 wt%)/SnNb_2_O_6_	[Co(bpy)_3_]^2+^	>420	0
6	Pt(0.3 wt%)/SnNb_2_O_6_	[Co(phen)_3_]^2+^	>420	0
7	Pt(0.3 wt%)/SrTiO_3_:Rh	[Co(bpy)_3_]^2+^	>420	12
8	Pt(0.3 wt%)/SrTiO_3_:Rh	[Co(phen)_3_]^2+^	>420	9.5
9	Ru(0.7 wt%)/SrTiO_3_:Rh	[Co(bpy)_3_]^2+^	>420	16
10	Ru(0.7 wt%)/SrTiO_3_:Rh	[Co(phen)_3_]^2+^	>420	12
11	Ru(0.7 wt%)/SrTiO_3_:Rh	Co^2+^	>420	0.5

^a)^ Reaction conditions: catalyst, 0.1 g; starting reacting solution, 120 mL aqueous solution of [Co(bpy)_3_]SO_4_ or [Co(phen)_3_]Cl_2_ with 0.5 mmol L^−1^ (pH = 7). Top irradiated cell with Xe‐arc lamp.

**Table 2:** Overall water splitting using Ru/SrTiO_3_:Rh as HEP with various OEPs in [Co(bpy)_3_]^3+/2+^ and [Co(phen)_3_]^3+/2+^ systems. Adapted with permission.^54^ Copyright 2013, American Chemical Society

Entry^a)^	OEPs	Starting reactant solution	Activity [µmol h^−1^]	
			H_2_	O_2_
1	WO_3_	[Co(bpy)_3_]SO_4_	14	0.5
2	WO_3_	[Co(phen)_3_]Cl_2_	15	0.4
3	TiO_2_:Cr,Sb	[Co(bpy)_3_]SO_4_	3.0	0.8
4	TiO_2_:Cr,Sb	[Co(phen)_3_]Cl_2_	1.3	0.7
5	BiVO_4_	[Co(bpy)_3_]SO_4_	10	4.8
6	BiVO_4_	[Co(phen)_3_]Cl_2_	7.9	3.5
7	BiVO_4_	CoSO_4_	1.0	0.2
8	BiVO_4_	2,2′‐bipyridine	3.2	0.6
9	BiVO_4_	1,10‐phenanthroline	5.5	0.5

^a)^ Reaction conditions: catalyst, 0.1 g; starting reacting solution, 120 mL aqueous solution of [Co(bpy)_3_]SO_4_ or [Co(phen)_3_]Cl_2_ with 0.5 mmol L^−1^ (pH = 7). Top irradiated cell with Xe‐arc lamp.

**Figure 12:**
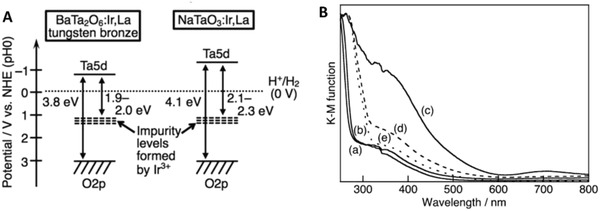
Electronic configuration of various doped metal oxide. A) Band structure for Ir and La co‐doped BaTa_2_O_6_. B) UV–vis DRS of ATa_2_O_6_:Ir,La where A is equal to a) Ca, b) Sr, and c–e) Ba. Samples of (a–c) were prepared via borate‐flux method whereas (d,e) were prepared using NaCl‐flux and SSR methods. Adapted with permission.[qv: 37d] Copyright 2017, Royal Society of Chemistry.

**Figure 13:**
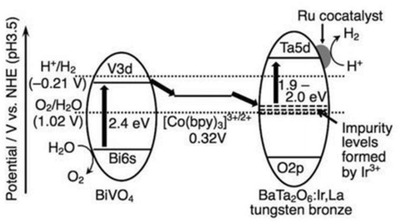
Z‐scheme water splitting of doped metal oxide in [Co(bpy)_3_]^3+/2+^ redox system. Mechanism of water splitting reaction containing Ru‐doped BaTa_2_O_6_:Ir,La as HEP, BiVO_4_ as OEP in solution with [Co(bpy)_3_]SO_4_ complex under visible light. Adapted with permission.[qv: 37d] Copyright 2017, Royal Society of Chemistry.

In a recent study of Ir and La ions‐doped BaTa_2_O_6_, it was declared that the intrusion of foreign atoms into the lattice of metal oxide will induce the formation of a new hybridized state, as shown in **Figure** **12**a. An insight on the intrinsic properties of these newly formed impurity states can be examined through UV–vis DRS as depicted in Figure 12B, in which all the doped samples exhibited an absorption tail in the visible region. The enhancement of visible light absorption after doping is attributed to the band trailing effect associated with the distortion in lattice. In this context, the doped metal ions are mainly in trivalent state which contributes to a shallow midgap level. As a result, the impurity levels can act as electron trapping centers which allow BaTa_2_O_6:_Ir,La to render photon absorption up to 640 nm. The corresponding overall water splitting mechanism in [Co(bpy)_3_]^3+/2+^ redox system is displayed in **Figure** **13**. It can be clearly visualized that the new hybridized impurity level can act as a recombination center to receive electrons from CB of BiVO_4_ via Co^3+^‐complex. Thus, the cascade electron transfer pathway within the Z‐scheme system is facilitated, culminating to stoichiometric evolution of H_2_ and O_2_ with large turnover number.

As of recent, vanadate redox system, which comprises of VO_2_^+^/VO^2+^ ionic couple, is employed as an electron mediator to govern PS‐A/D‐PS Z‐schematic reaction.[qv: 37b] It is worth noting that the redox cycle between V^5+^ and V^4+^ is competent in relaying electron according to Equation (12). However, pH has a great influence on the standard potential of V^5+/4+^ complex. In this regard, the redox potential of V^5+/4+^ complex was measured to be +0.7 and +1.0 V versus NHE at pH 3.8 and 1.7, respectively. According to **Table** **3**, the photocatalytic O_2_ evolution of TiO_2_ in a solution containing V^5+^ ions decreased when the pH increased from pH 1.7 to pH 6.5 and 10.9. The enhanced performance of water oxidation by TiO_2_ at pH 1.7 is attributed to presence of VO_2_^+^ ions at lower pH, which serves as an effective reductant for semiconductors in photocatalytic reaction. In whole, this section provides a discerning understanding on the properties of various redox couples in constructing PS‐A/D‐PS system

(12) $$4{\text{VO}}_{2}{}^{+}+\;4{\text{H}}^{+}\;4{\text{VO}}^{2+}+\;{\text{O}}_{2}+2{\text{H}}_{2}\text{O}$$

**Table 3:** Dependency of pH on vanadate redox system. O_2_ evolution from solution containing V^5+^ ions and TiO_2_. Adapted with permission.[qv: 37b] Copyright 2017, American Chemical Society

Entry^a)^	Photocatalysts	Initial pH	Incident light [nm]	O_2_ Evolution rate [µmol h^−1^]
1	TiO_2_	1.7	>300	64
2	TiO_2_	6.5	>300	2
3	TiO_2_	10.9	>300	<1

^a)^ Reaction conditions: catalyst, 0.4 g; aqueous 2 × 10^−3^ M V^5+^ solution; 300 mL; 300 W Xe lamp.

**Figure 14:**
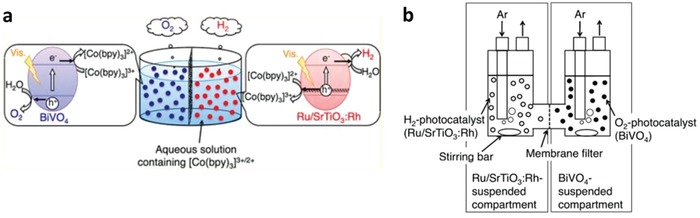
Separated gas evolution of H_2_ and O_2_ from photocatalytic overall water splitting. a,b) Schematic illustration of on‐site separation of H_2_ and O_2_ gases from two‐compartment PS‐A/D‐PS system. Adapted with permission.^54^ Copyright 2013, American Chemical Society.

With the capability of ionic redox pairs in driving reversible cycles for PS‐A/D‐PS Z‐scheme, incessant studies have been carried out to construct overall water splitting system for harvesting H_2_ fuel. The summary of PS‐A/D‐PS Z‐scheme heretofore was tabulated in **Table** **4**. However, one of the biggest weaknesses with redox‐mediated system is their pH dependency imputed to the limitation of ionic pairs. As previously mentioned, IO_3_^−^/I^−^ can only work efficiently at a more basic condition with pH more than 9 ascribed to the formation of inactive I_3_^−^ under lower pH. Whereas for Fe^3+^/Fe^2+^ redox cycle, the system is only stable at pH lower than 2.5 due to the precipitation of Fe(OH)_3_ from Fe^3+^ under higher pH. This greatly restricts the application of redox mediator at a wide pH range. On top of that, the presence of redox mediator will absorb light to a certain extent which reduces the available photon for the photocatalysis reaction. With this in mind, development of redox mediator‐free Z‐scheme is imperative to surmount the problems from PS‐A/D‐PS system. Nevertheless, PS‐A/D‐PS system also provides a prominent platform to mimic natural photosynthesis and demonstrates remarkable results for overall water splitting. In addition, separation of produced H_2_ and O_2_ gas on site can be achieved in PS‐A/D‐PS system with a suitable reactor as shown in **Figure** **14**. The presence of membrane filter in two‐compartment cell can allow the diffusion of ions while retaining the evolved gas in separated columns. Hence, selective evolution of H_2_ and O_2_ gases on separated cells can be accomplished, which poses their upper hand as compared to other powder suspension system.

**Table 4:** Summary of PS‐A/D‐PS system for water splitting

Entry	PS I (available λ)	PS II (available λ)	Mediator	Light source	Efficiency	Ref.
1	Pt/TiO_2_ anatase (<380 nm)	TiO_2_ rutile (<410 nm)	IO_3_^−^/I^−^	400 W Hg lamp (λ > 300 nm)	H_2_: 180 µmol h^−1^; O_2_: 90 µmol h^−1^	20
2	Pt/SrTiO_3_:Cr,Ta (<550 nm)	Pt/WO_3_ (<460 nm)	IO_3_^−^/I^−^	300 W Xe lamp (λ > 420 nm)	AQY: 0.1% (420.7 nm)	55
3	Pt/TaON (<520 nm)	Pt/WO_3_ (<460 nm)	IO_3_^−^/I^−^	300 W Xe lamp (λ > 420 nm)	AQY: 0.4% (420 nm)	56
4	Pt/BaTaO_2_N (<660 nm)	Pt/WO_3_ (<460 nm)	IO_3_^−^/I^−^	300 W Xe lamp (λ > 420 nm)	AQY: 0.1% (420–440 nm)	35
5	Pt/ZrO_2_/TaON (<520 nm)	Pt/WO_3_ (<460 nm)	IO_3_^−^/I^−^	300 W Xe lamp (420 < λ < 800 nm)	AQY: 6.3% (420.5 nm)	[qv: 36a]
6	Pt/BaZrO_3_/BaTaO_2_N (<660 nm)	Pt/WO_3_ (<460 nm)	IO_3_^−^/I^−^	300 W Xe lamp (420 < λ <800 nm)	AQY: 0.6% (420–440 nm)	57
7	Pt/Sm_2_Ti_2_S_2_O_5_ (<650 nm)	TiO_2_ rutile (<410 nm)	IO_3_^−^/I^−^	450 W Hg lamp (λ > 300 nm)	H_2_: 45 µmol h^−1^; O_2_: 16 µmol h^−1^	58
8	Pt/g‐C_3_N_4_ (<442 nm)	Pt/WO_3_ (<460 nm)	IO_3_^−^/I^−^	300 W Xe lamp (λ > 395 nm)	H_2_: 74 µmol h^−1^; O_2_: 37 µmol h^−1^	59
9	Pt/MgTa_2_O_6−_ *_x_*N*_y_*/TaON (<570 nm)	PtO*_x_*/WO_3_ (<460 nm)	IO_3_^−^/I^−^	300 W Xe lamp (λ > 420 nm)	AQY: 6.8% (420 nm)	60
10	Pt‐IrO_2_/Sm_2_Ti_2_S_2_O_5_ (<590 nm)	PtO*_x_*/H‐Cs‐WO_3_ (<460 nm)	IO_3_^−^/I^−^	300 W Xe lamp (λ > 420 nm)	H_2_: 40.6 µmol h^−1^; O_2_: 16.1 µmol h^−1^	61
11	Ru/SrTiO_3_:Rh (<520 nm)	RuO_2_/TiO_2_:Ta,N (<510 nm)	IO_3_^−^/I^−^	Xe lamp (420 < λ < 800 nm)	H_2_: 1.3 µmol h^−1^; O_2_: 0.5 µmol h^−1^	52
12	Ru/SrTiO_3_:Rh (<520 nm)	WO_3_ (<460 nm)	Fe^3+^/Fe^2+^	300 W Xe lamp (λ > 420 nm)	H_2_: 416 µmol h^−1^; O_2_: 197 µmol h^−1^	[qv: 37c]
13	Ru/SrTiO_3_:Rh (<520 nm)	BiVO_4_ (<520 nm)	Fe^3+^/Fe^2+^	300 W Xe lamp (λ > 420 nm)	AQY: 4.2% (420 nm); STH: 0.1%	42
14	Ru/SrTiO_3_:Rh (<520 nm)	IrO*_x_*/SrTiO_3_:Rh,Sb (<560 nm)	Fe^3+^/Fe^2+^	300 W Xe lamp (λ > 420 nm)	H_2_: 3 µmol h^−1^; O_2_: 1.4 µmol h^−1^	62
15	Pt/g‐C_3_N_4_ (<442 nm)	BiVO_4_ (<520 nm)	Fe^3+^/Fe^2+^	300 W Xe lamp (λ > 395 nm)	H_2_: 15 µmol h^−1^; O_2_: 8 µmol h^−1^	59
16	Ru/SrTiO_3_:Rh (<520 nm)	RuO_2_/TiO_2_:Ta,N (<510 nm)	Fe^3+^/Fe^2+^	Xe lamp (420 < λ < 800 nm)	STH: 0.021%	52
17	Ru/SrTiO_3_:Rh (<520 nm)	Bi_4_TaO_8_Cl (<480 nm)	Fe^3+^/Fe^2+^	Xe lamp (420 < λ < 800 nm)	AQY: 0.9% (420 nm)	63
18	Ru/SrTiO_3_:Rh (<520 nm)	Bi_4_TaO_8_Br (<500 nm)	Fe^3+^/Fe^2+^	300 W Xe lamp (λ > 420 nm)	H_2_: 3 µmol h^−1^; O_2_: 1.3 µmol h^−1^	64
19	Ru/SrTiO_3_:Rh (<520 nm)	RuO_2_/Bi_4_NbO_8_Cl (<490 nm)	Fe^3+^/Fe^2+^	300 W Xe lamp (λ > 400 nm)	AQY: 1.3% (420 nm)	48
20	Ru/SrTiO_3_:Rh (<520 nm)	BiVO_4_ (<520 nm)	[Co(bpy)_3_]^3+/2+^	300 W Xe lamp (λ > 420 nm)	H_2_: 10 µmol h^−1^; O_2_: 4.8 µmol h^−1^	54
21	Ru/SrTiO_3_:Rh (<520 nm)	WO_3_ (<460 nm)	[Co(phen)_3_]^3+/2+^	300 W Xe lamp (λ > 420 nm)	H_2_: 15 µmol h^−1^; O_2_: 0.4 µmol h^−1^	54
22	Ru/SrTiO_3_:Rh (<520 nm)	Fe‐H‐Cs‐WO_3_ (<460 nm)	VO_2_^+^/VO^2+^	300 W Xe lamp (λ > 420 nm)	STH: 0.03%	[qv: 37b]
23	Pt/NiS‐La_5_Ti_2_AgS_2_O_7_ (<650 nm)	Fe‐H‐Cs‐WO_3_ (<460 nm)	IO_3_^−^/I^−^	300 W Xe lamp (λ > 420 nm)	AQY: 0.12% (420 nm)	65

### 3.2 PS‐C‐PS System (Second Generation)

#### 3.2.1 Mechanism of Electron Mediator

**Figure 15:**
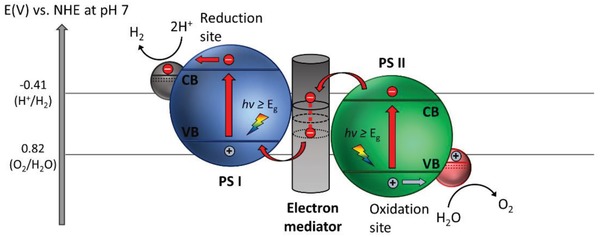
Schematic band energy diagram of PS‐C‐PS Z‐scheme system.

The inevitable drawbacks from PS‐A/D‐PS Z‐scheme, such as backward reaction and shielding effect, have impeded further application of such redox‐mediated system. This has prompted the investigation on the development of redox‐mediator‐free system. In fact, the two‐step photoexcitation system can be devised in all‐solid‐state without the need of ionic pairs. A recreation of the system can be witnessed in all‐solid‐state PS‐C‐PS in which A/D pair is substituted by a conductor (C) as the electron mediator, which is shown in **Figure** **15**. The intimate contact between the photocatalysts and mediator allows the photogenerated electrons from PS II to be readily recombined with the photogenerated holes from PS I through a low contact resistance interface, namely Ohmic contact.^24^ Unlike the inefficient redox shuttle, solid‐state mediator renders a more promising electron relaying ability by providing interparticle electron transfer which greatly trims the distance of electron flow from PS II to PS I.^66^ Besides, backward reaction and shielding effect can be perfectly prevented due to the absence of A/D pair. On top of that, PS‐C‐PS system is also suitable for both gas phase and liquid phase reaction. The potential candidates to serve as an efficient solid‐state mediator can be generally classified into two major categories, i.e., metal (Au, Ag, Ir, Cd, W, Ni, etc.) and conductive carbon (graphene, CNTs, CQDs, etc.), attributed to their superior electronic properties in facilitating electron transfer.^66^, ^67^ With the employment of highly conductive materials in between PS systems, vectorial electron transfer can be accomplished as mean for enhancing the isolation of active carriers. The implication of various conductors in PS‐C‐PS system will be discussed henceforth.

#### 3.2.2 Metallic Electron Mediators

##### Noble Metals

**Figure 16:**
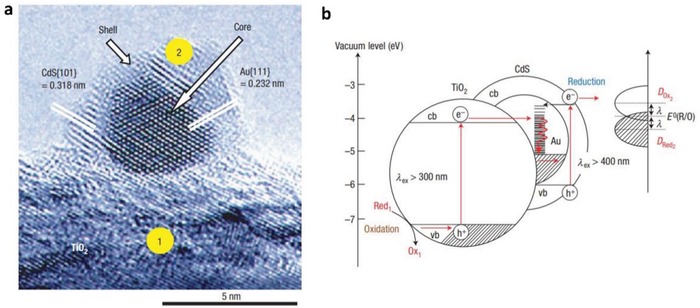
All‐solid‐state Z‐scheme with Au as electron mediator. a) HRTEM image and b) schematic illustration of charge transfer in CdS/Au/TiO_2_. Adapted with permission.^23^ Copyright 2006, Nature Publishing Group.

**Figure 17:**
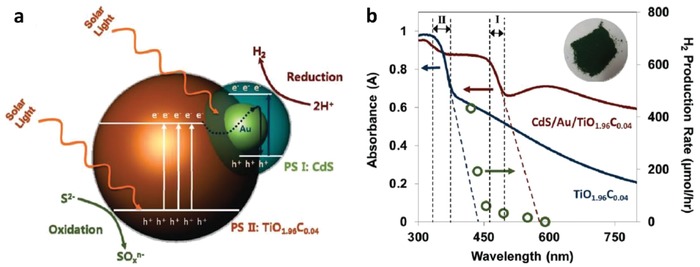
a) Charge transfer mechanism of CdS/Au/TiO_1.96_C_0.04_. b) UV–vis of TiO_1.96_C_0.04_ and CdS/Au/TiO_1.96_C_0.04_. Adapted with permission.^68^ Copyright 2011, American Chemical Society.

In 2006, the construction of CdS/Au/TiO_2_ ternary structure by Tada et al. marked the beginning of all‐solid‐state Z‐scheme system in achieving photocatalytic water splitting.^23^ Ever since, solid‐state mediator has emerged as a rising star and received keen interest from worldwide over the former redox mediator for hosting Z‐schematic water splitting system. The three‐component CdS/Au/TiO_2_ nanojunction that is synthesized through photochemical deposition–precipitation method is able to seize the large work function of Au nanoparticles to transfer electron. The unique type of Z‐scheme structure highlights the importance of ingenious arrangement of semiconductors in nanoscale. As depicted in **Figure** **16**a, a core–shell configuration of Au/CdS was deposited on top of TiO_2_ which resembles an intimate Z‐scheme relationship between CdS (101)||Au (111)||TiO_2_ (101) orientated nanostructure. The ternary structure demonstrated a higher photocatalytic activity in stark contrast to the two‐component systems of either Au/CdS or TiO_2_/CdS, attributed to the facilitated cascade electron transfer between CdS and TiO_2_ via Au electron mediator (Figure 16b). Later on, C‐doped TiO_2_ (TiO_1.96_C_0.04_) was studied by Yun et al. to replace TiO_2_ in the Z‐scheme system of CdS and Au, as displayed in **Figure** **17**a.^68^ Unlike TiO_2_ with large band, TiO_1.96_C_0.04_ displayed a larger extend of light absorption close to 440 nm, which is corresponded to bandgap of 2.6 eV. It is worth noting that the presence of two shoulders in UV–vis DRS of CdS/Au/ TiO_1.96_C_0.04_ (Figure 17b) matches with the absorption profile of TiO_1.96_C_0.04_ (region II) and CdS (region I), which corroborate the co‐existence of both semiconductors in the Z‐scheme system. As a result, CdS/Au/TiO_1.96_C_0.04_ exhibited an exceptional high H_2_ evolution rate of 433.2 µmol h^−1^ in the presence of Na_2_S/Na_2_SO_3_ sacrificial condition, which is about 4.7‐fold enhancement as compared to CdS/Au/TiO_2_. Besides, the AQY of H_2_ production was measured to be 23.6% under monochromatic light of 420 nm. Even though the water splitting reaction occurs with the aids of sacrificial reagents, the striking idea of all‐solid‐state Z‐scheme has evoked considerable attention toward its development in water splitting.

**Figure 18:**
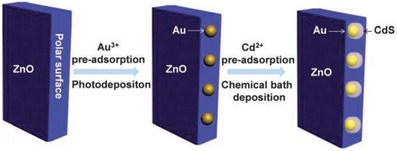
Synthetic protocol of preparation of CdS/Au/ZnO Z‐scheme structure via two‐step assembly method. Adapted with permission.[qv: 67a] Copyright 2013, Royal Society of Chemistry.

**Figure 19:**
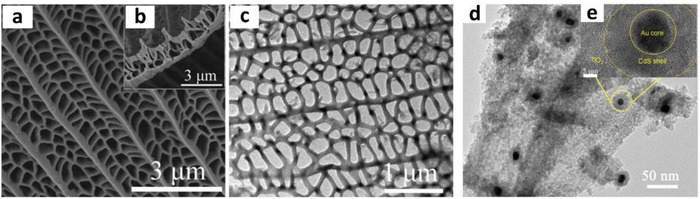
Geometry of PS‐C‐PS system that resembles butterfly wings. a,b) FESEM images of WA‐TiO_2_. c) TEM image of WA‐TiO_2_. d,e) TEM images of CdS/Au/WA‐TiO_2_. Adapted with permission.^69^ Copyright 2013, Elsevier.

Hitherto, there has been an incessant endeavor in constructing PS‐C‐PS Z‐scheme system with control of geometry architecture. The fabrication of flower‐like spheres of ZnO as a fertile template to load Au core with CdS shell highlights the significance of arrangement of nanoparticles in order to enhance the solar light harvesting with improved charge separation efficiency and remarkable large specific surface area.[qv: 67a] As shown in **Figure** **18**, core/shell Au/CdS was selectively deposited onto the negatively charged polar surface of ZnO (0002) due to the difference in affinity. The improved interfacial transfer of charge carriers resulted in an augmentation of photocatalytic H_2_ evolution under Na_2_S/Na_2_SO_3_ solution, which is ≈4.5 times higher than the corresponding CdS/ZnO without Au as the mediator. Interestingly, wing‐architecture TiO_2_ (WA‐TiO_2_) prepared by immersion–calcination route resembles the antireflection properties of butterfly wings, as shown in **Figure** **19**a,b.^69^ Core/shell Au/CdS was subsequently loaded on WA‐TiO_2_ using a similar two‐step deposition method, forming a Z‐scheme heterostructure (Figure 19d,e). According to the simulated results, light absorption of WA‐TiO_2_ has been greatly enhanced due to its scale architecture which reduced the reflection of UV light by 40%. As a result, photocatalytic H_2_ production of CdS/Au/WA‐TiO_2_ has been improved by about two times as compared to Z‐scheme system which employed flat TiO_2_. This finding elucidates the significance of geometry architecture in photocatalysts to improve the Z‐scheme performance.

**Figure 20:**
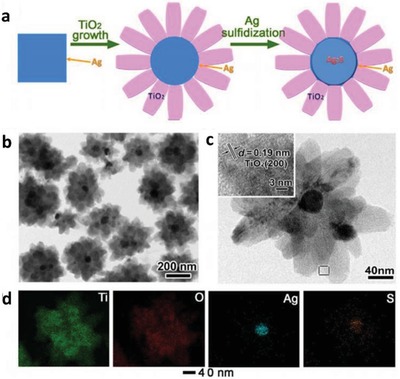
PS‐C‐PS system with in situ generation of conductor. a) Synthetic protocol of Ag_2_S/Ag/TiO_2_. b,c) FESEM and HRTEM images of Ag_2_S/Ag/TiO_2_ and d) the corresponding EDX mapping. Adapted with permission.^70^ Copyright 2015, Springer.

**Figure 21:**
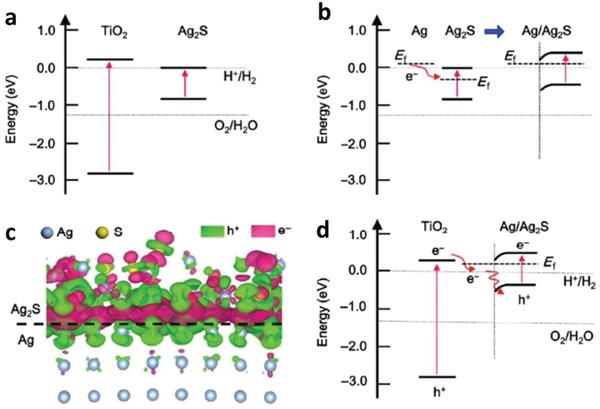
a) Computed band structure of bulk TiO_2_ and Ag_2_S. b) Schematic of electron flow in Ag/Ag_2_S system. c) Computed differential charge distribution at interface of Ag_2_S and Ag. d) Schematic illustration of Z‐scheme formation for Ag_2_S/Ag/TiO_2_. Adapted with permission.^70^ Copyright 2015, Springer.

Apart from controlling the geometry of Z‐scheme architecture, PS‐C‐PS system can allow in situ generation of conductor from the photocatalysts if the material of the mediator is the same as PS I or II. Ag_2_S/Ag/TiO_2_ from the studies by Li et al. is one of the examples with interfacial Ag serving as the in situ mediator for the Z‐scheme system, as shown in **Figure** **20**.^70^ Typically, TiO_2_ was firstly grown on Au nanocubes forming a core/shell structured Ag/TiO_2_, which underwent sulfurization of Ag core into Ag_2_S. The resulting product confers Z‐scheme configuration of PS I‐C‐PS II with vectorial electron transfer, as delineated in **Figure** **21**. As a result, the in situ formed metallic mediator is firmly embedded onto the surface of photocatalysts, which can create a germane interaction between conductor and photosystems and, at the same time preventing the exfoliation of mediator. According to first‐principle calculations, the strong interaction between Ag and Ag_2_S can induce the injection of electrons from Ag to Ag_2_S through the space charge region.^70^ Consequently, the Fermi level of Ag_2_S was shifted upward by ≈0.47 eV. However, the interaction of Ag and TiO_2_ is milder as compared to Ag_2_S and hence, it is less likely for electrons to flow from Ag to TiO_2_. Thus, electrons are dictated from TiO_2_ (CB) to Ag_2_S (VB) via the interconnected Ag, as displayed in Figure 21d. This bestows a new approach to synthesize Z‐scheme photocatalysts with high efficiency for photocatalytic water splitting.

##### Transition Metals

**Figure 22:**
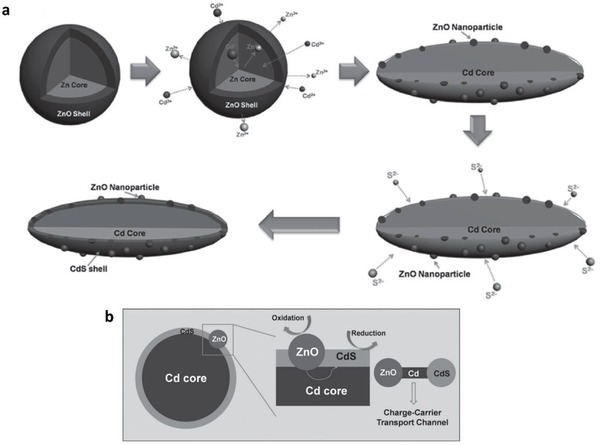
a) Synthetic protocol and b) charge transfer profile of CdS/Cd/ZnO Z‐scheme system. Adapted with permission.^66^ Copyright 2012, Wiley.

Other than rare and expensive noble metal, low cost transition metal is amenable to serve as a conductor in PS‐C‐PS Z‐scheme system. In a study employing Cd as an electron mediator, it was found that the presence of this metallic core can efficiently facilitate the charge transfer between CdS and ZnO as PS I and II.^66^ As postulated in **Figure** **22**, CdS/Cd/ZnO ternary heterostructure was prepared using a two‐step process which involves the formation of Cd core by penetrating into ZnO. Due to the facile evaporation of metal Zn from the interior of ZnO, the Zn core was replaced by Cd while retaining a thin layer ZnO shell. The ZnO shell was then ruptured into ZnO nanoparticles and attached to the Cd core. Further sulfurization process leads to the formation of CdS shell which covers Cd core together with the ZnO nanoparticles. In this context, the Cd core behaves as a charge‐transporting channel between CdS and ZnO which improves the charge isolation and prolongs the lifetime of photogenerated charge carrier. As a result, Pt‐loaded CdS/Cd/ZnO Z‐scheme system demonstrated photocatalytic H_2_ evolution rate of 1.92 mmol h^−1^ under 300 W Xe lamp irradiation, which is ≈5.1 times enhancement over CdS/ZnO nanostructure without a Cd core. The development of such transition metal‐aided Z‐scheme system is very similar to the in situ formation of conductor as discussed previously in the Ag‐mediated PS‐C‐PS system. This highlights the importance of metallic core in assisting facile charge transport and improving the photocatalytic performance.

**Figure 23:**
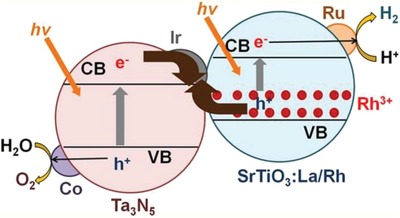
Schematic illustration of charge transfer profile for Ru/SrTiO_3_:La,Rh‐Ir‐CoO*_x_*/Ta_3_N_5_ Z‐scheme system. Adapted with permission.^71^ Copyright 2014, American Chemical Society.

Iridium (Ir), being another transition metal that confers similar electronic properties as Pt, is competent for driving electron transfer within PS‐C‐PS Z‐scheme system. Wang et al. reported a Z‐scheme system comprising of Ru/SrTiO_3_:La,Rh as PS I and CoO*_x_*/Ta_3_N_5_ as PS II mediated by an Ir conductor.^71^ The developed Z‐scheme system is capable to perform photocatalytic water splitting without the use of any sacrificial reagent, which imminently manifested the efficiency of such nanostructure. As depicted in **Figure** **23**, cascade electron transfer profile occurs in which electrons from Ta_3_N_5_ (CB) was relayed to SrTiO_3_:La,Rh (VB) via Ohmic contact at Ir‐semiconductors interface. The presence of co‐doping of La and Rh ions into the lattice of SrTiO_3_ can substitute parts of the Ti^4+^ sites in SrTiO_3_ by inducing oxygen vacancies to compensate the higher valence states. As aforementioned, the existence of vacancy sites can lead to the formation of midgap states which reduce the bandgap of the semiconductor. Consequently, SrTiO_3_:La,Rh exhibited light absorption up to 700 nm. Visible‐light‐driven photocatalytic water splitting reaction of the Z‐schematic Ru/SrTiO_3_:La,Rh‐Ir‐CoO*_x_*/Ta_3_N_5_ system demonstrated remarkable activities of H_2_ and O_2_ evolution with measured AQY and STH of 1.1% and 0.037%, respectively.^71^

#### 3.2.3 Conductive Carbon Electron Mediators

##### Graphene

While the research on metallic conductor in PS‐C‐PS system is becoming more prevalent, nanocarbon‐based electron mediator is a relatively new addition to the family of Z‐scheme owing to the versatile tailoring and extraordinary electronic properties.^72^ Nanocarbon materials, for instance, graphene (2D), CNTs (1D), and CQDs (0D), are worth noting as promising candidates to facilitate vectorial electron transfer between PS I and II that underpins the Z‐scheme system. As is known, graphene is one of the functionalized carbon allotropes that features ballistic electron mobility and excellent electrical conductivity.^73^ The 2D layer arrangement of carbon atoms in honeycomb lattice of graphene can be exfoliated from graphite by chemical or physical means to fully harness the function of this nanocarbon. A single layer of highly oxidized graphene is also known as graphene oxide (GO). However, GO produced by acid treatment (such as modified Hummers' method) often suffers from defects and deteriorated electrical properties.^74^ In this context, GO is often being introduced in its reduced form, reduced graphene oxide or RGO, which is obtained via sequential oxidation–exfoliation–reduction route.^75^ However, it is noteworthy to mention that the degree of reduction in RGO will influence the hydrophilicity of the whole composite, which might cause difficulties for its dispersion in water.

**Figure 24:**
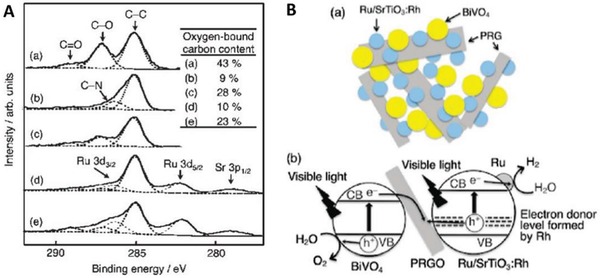
A) C 1s XPS spectra of a) GO, b) hydrazine‐reduced GO, c) PRGO/BiVO_4_, d) PRGO/SrTiO_3_:Rh, and e) Ru/SrTiO_3_:Rh‐(PRGO/BiVO_4_). B) Schematic illustration of charge transfer mechanism in Ru/SrTiO_3_:Rh‐(PRGO/BiVO_4_). Adapted with permission.^25^ Copyright 2011, American Chemical Society.

The first graphene‐mediated Z‐scheme system for photocatalytic water splitting was studied by Iwase et al., in which Ru/SrTiO_3_:Rh and BiVO_4_ were employed as PS I and II, respectively.^25^ In this work, RGO was incorporated into the Z‐scheme system through two reduction methods of GO, namely chemical reduction method using hydrazine and photoreduction. The degree of restoration of graphitic structure after reduction was determined by measuring the content of oxygen‐bounded carbon, as shown in **Figure** **24**a. GO reduced by hydrazine displayed the lowest oxygen‐bound carbon content (9%) ascribed to the strong reduction by chemical route. On the other hand, photoreduced GO (PRGO) was obtained through reduction of GO on either of Ru/SrTiO_3_:Rh or BiVO_4_. Owing to the more electronegative nature of Ru/SrTiO_3_:Rh, the reduction of GO on Ru/SrTiO_3_:Rh is stronger than that on BiVO_4_. Hence, the oxygen‐bound carbon content of PRGO/BiVO_4_ (28%) was found to be higher than that of PRGO/SrTiO_3_:Rh (10%), which indicates a lower restoration of graphitic structure. According to the overall water splitting activities in **Table** **5**, ternary Z‐scheme system with PRGO on BiVO_4_ (entry 8) demonstrated the highest photocatalytic gas evolution. Imputed to the strong hydrophobicity of RGO produced by hydrazine and photoreduction on Ru/SrTiO_3_:Rh, the photocatalysts were found to be immiscible in water. Thus, it is crucial to obtain a balance between the degree of reduction of GO and the hydrophilic nature of the composites in water. The presence of PRGO can facilitate the cascade electron transfer from BiVO_4_ to Ru/SrTiO_3_:Rh, as shown in Figure 24B.

**Table 5:** Photocatalytic performance of PRGO‐based Z‐scheme. Overall water splitting activities of Ru/SrTiO_3_:Rh‐BiVO_4_ system with and without RGO as electron mediator under visible light. Adapted with permission.^25^ Copyright 2011, American Chemical Society

Entry^a)^	PS I	Mediator	PS II	pH	Activities[µmol h^−1^]	
					H_2_	O_2_
1	Ru/SrTiO_3_:Rh	–	–	3.5	0.9	0
2	Ru/SrTiO_3_:Rh	PRGO(Ru/SrTiO_3_:Rh)	–	3.5	0.9	0
3	–	–	BiVO_4_	3.5	0	0
4	–	PRGO(BiVO_4_)	BiVO_4_	3.5	0	0
5	Ru/SrTiO_3_:Rh	–	BiVO_4_	3.5	3.7	1.9
6	Ru/SrTiO_3_:Rh	–	BiVO_4_	7.0	0.8	0.5
7	Ru/SrTiO_3_:Rh	PRGO(Ru/SrTiO_3_:Rh)	BiVO_4_	3.5	1.4	0.6
8	Ru/SrTiO_3_:Rh	PRGO(BiVO_4_)	BiVO_4_	3.5	11	5.5
9	Ru/SrTiO_3_:Rh	PRGO(BiVO_4_)	BiVO_4_	7.0	1.1	0.6
10	Ru/SrTiO_3_:Rh	N_2_H_4_‐RGO	BiVO_4_	3.5	4.8	2.3

^a)^ Reaction conditions: catalyst, 0.3 g; 120 mL of water adjusted by H_2_SO_4_; 300 W Xe lamp.

For years, metal chalcogenides have gained explosive popularity as efficient HEPs attributed to their narrow bandgap and suitable relative band edge for H_2_ production.^76^ A series of metal chalcogenides, particularly sulfides, were investigated by Iwashina et al. in Z‐schematic water splitting with rutile TiO_2_ as OEP and RGO as mediator.^77^ As depicted in **Table** **6**, Cu‐based ternary metal dichalcogenides (CuGaS_2_ and CuInS_2_) loaded with Pt demonstrated stoichiometric evolution of H_2_ and O_2_ when coupled with TiO_2_ in RGO‐mediated Z‐scheme system. As a stark contrast, Zn‐ and Ag‐based sulfides (entry 1–5) only show sluggish H_2_ evolution without O_2_ imputed to the gradual photocorrosion. Besides, the presence of RGO would propel the vectorial electron transfer between CuInS_2_ and TiO_2_. As shown in entry 10 of Table 6, the photocatalytic water splitting activity was drastically reduced with the absence of RGO. Furthermore, two‐component systems (entries 12 and 13) exhibited low activities of gas evolution as compared to the ternary systems. Thus, the synergistic combination of PS I, II and mediator is crucial in conferring feasible thermodynamics requirements for overall water splitting. Moreover, the incorporation of Pt onto HEP was essential to diminish the kinetic barrier for H_2_ production, in which the performance of Z‐scheme system without Pt was severely reduced with nonstoichiometric evolution of H_2_ and O_2_ gas as observed (entry 11).

**Table 6:** Photocatalytic performance of metal sulfides in RGO‐based Z‐scheme. Overall water splitting activities of RGO‐mediated Z‐scheme system with various metal sulfides and rutile TiO_2_. Adapted with permission.^77^ Copyright 2015, American Chemical Society

Entry^a)^	PS I	Mediator	PS II	Activities [µmol h^−1^]	
				H_2_	O_2_
1	Pt/ZnS	RGO	TiO_2_	0.8	0
2	Pt/AgGaS_2_	RGO	TiO_2_	12.5	0
3	Pt/AgInS_2_	RGO	TiO_2_	0.3	0
4	Pt/Ag_2_ZnGeS_4_	RGO	TiO_2_	6.1	0
5	Pt/Ag_2_ZnSnS_4_	RGO	TiO_2_	0.4	0
6	Pt/CuGaS_2_	RGO	TiO_2_	19.8	10.3
7	Pt/CuInS_2_	RGO	TiO_2_	9.9	4.5
8	Pt/Cu_2_ZnGeS_4_	RGO	TiO_2_	17.4	7.8
9	Pt/Cu_2_ZnSnS_4_	RGO	TiO_2_	6.3	2.9
10	Pt/CuGaS_2_	–	TiO_2_	1.4	0.3
11	CuGaS_2_	RGO	TiO_2_	6.9	0.8
12	Pt/CuGaS_2_	RGO	–	0.2	0
13	–	RGO	TiO_2_	0	0

^a)^ Reaction conditions: catalyst, 0.05 g each; 120 mL of water adjusted by H_2_SO_4_; 300 W Xe lamp.

**Figure 25:**
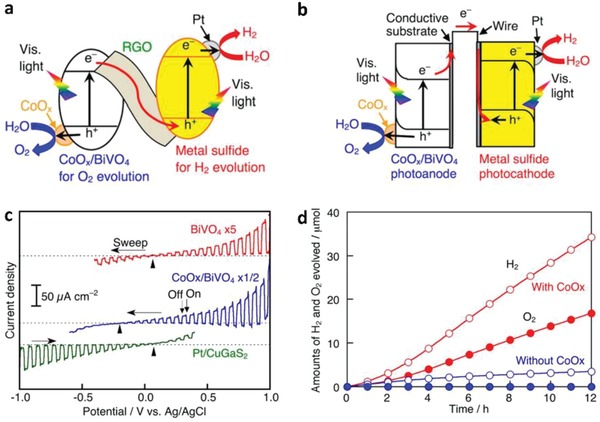
Charge transfer mechanism of Pt/metal sulfides‐CoO*_x_*/BiVO_4_ in a) RGO‐mediated Z‐scheme and b) PEC. c) Current‐potential curves of BiVO_4_, CoO*_x_*/BiVO_4_, and Pt/CuGaS_2_. d) Overall water splitting of Pt/CuGaS_2_‐RGO‐BiVO_4_ Z‐scheme system with and without CoO*_x_* under visible light. Adapted with permission.^78^ Copyright 2016, American Chemical Society.

In another investigation of Pt/CuGaS_2_‐RGO‐BiVO_4_ Z‐scheme system by Iwase et al., p‐type and n‐type semiconductors were connected through an electron mediator and that resembles the electron flow in PEC cell (**Figure** **25**b).^78^ In this system, RGO can be regarded as an electron relaying channel that shuttles electron transfer from n‐type BiVO_4_ to p‐type Pt/CuGaS_2_ without any external bias, as shown in Figure 25a. Besides, Figure 25c shows the cathodic and anodic photocurrent nature of Pt/CuGaS_2_ and BiVO_4_, respectively. One step further can be witnessed when CoO*_x_* co‐catalysts were loaded on BiVO_4_, in which the on‐set potential of BiVO_4_ is dictated toward the negative direction attributed to the facilitation of water oxidation. The overlapping of potential from Pt/CuGaS_2_ and CoO*_x_*/BiVO_4_ suggests that the electron can flow without any external bias. In other words, RGO‐mediated Pt/CuGaS_2_‐CoO*_x_*/BiVO_4_ system can demonstrate cascade electron transfer from PS II to I, resulting in visible‐light‐driven overall water splitting with stoichiometric evolution of H_2_ (3.5 µmol h^−1^) and O_2_ (1.7 µmol h^−1^) gas as shown in Figure 25d.

**Figure 26:**
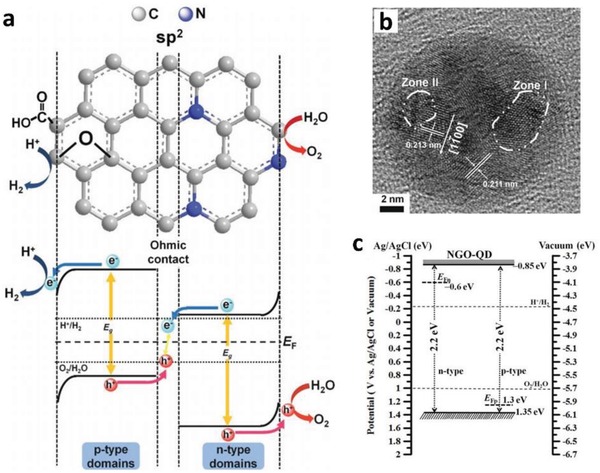
Heteroatoms doped GO in Z‐scheme. a) Schematic illustration of charge transfer mechanism of NGO‐QDs. b) Representative HRTEM image of NGO‐QDs. c) Energy band energy diagram of NGO‐QDs. Adapted with permission.^80^ Copyright 2014, Wiley.

Heteroatoms (N, B, P and S) doping into lattice of graphene is a relatively new introduction to the nanocarbon family.^79^ The intrusion of foreign atom into the graphitic framework could significantly alter the electronic and physicochemical properties of graphene. Yeh et al. reported the coexistence of both p‐type and n‐type conductivities in N‐doped graphene quantum dots (NGO‐QDs) which allows a single doped material to exhibit Z‐scheme electronic configuration (**Figure** **26**a).^80^ Due to the quantum confinement effect, bandgap opening occurs on GO‐QDs which initially contain zero bandgap since π and π* orbitals dispersion of pristine graphene touch in the Brillouin zone. GO derived from surface modification of graphite will introduce oxygenated species that confer p‐type electronic natures ascribed to the more electronegative behavior of O than C atom. Further doping of N onto GO‐QDs will introduce n‐type semiconductor characteristic, which is beneficial for water oxidation. The resultant NGO‐QDs simultaneous bestow both p‐type and n‐type electronic properties, resembling an internal Z‐scheme structure. Figure 26b displays the HRTEM image of NGO‐QDs, in which two zones with different lattice arrangement can be observed. According to Figure 26a,c, NGO‐QDs convene both p‐type and n‐type domains connected by undoped sp^2^ region, which function as an internal conductor that facilitates vectorial electron transfer via Ohmic contact. The p‐type domain contains more electronegative band structure while n‐type domain is more electropositive. Hence, each domain can function effectively as HEP and OEP, respectively. Consequently, NGO‐QDs demonstrated efficient overall water splitting performance under visible light with stoichiometric evolution of H_2_ and O_2_ gas.^80^ These findings present an avenue to tailor the geometry architecture of PS‐C‐PS Z‐scheme using graphene as an electron mediator that is competent to govern overall water splitting without any sacrificial reagent.

##### Other Nanocarbons

**Figure 27:**
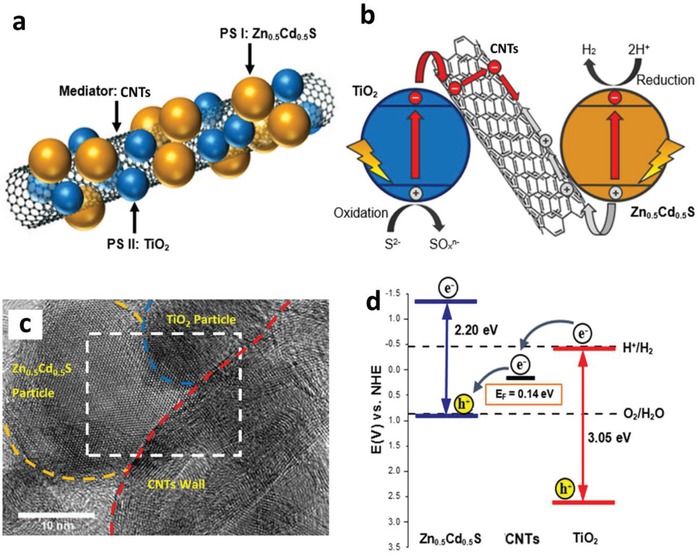
Schematic illustration of a) Zn_0.5_Cd_0.5_S‐CNTs‐TiO_2_ and b) its charge transfer mechanism. c) HRTEM image of Zn_0.5_Cd_0.5_S‐CNTs‐TiO_2_. d) Band energy diagram of Zn_0.5_Cd_0.5_S‐CNTs‐TiO_2_. Adapted with permission.[qv: 17a] Copyright 2017, Elsevier.

In addition to graphene discussed earlier, other nanocarbon materials such as CNTs and CQDs are also competent to drive vectorial electron transfer within Z‐scheme system. CNTs, a dimensionally confined sp^2^ bonded carbon allotrope that features versatile tailoring and excellent intrinsic properties, are worth noting as potential candidate for hosting Z‐scheme composites.^81^ Owing to the excellent conduction ability of CNTs, electrons can travel up to a remarkable 1 × 10^5^ cm^2^ V^−1^ s^−1^ mobility at room temperature.^82^ Besides, the excellent mechanical properties and a theoretical large surface area render the suitability of CNTs to be used as a support template for the formation of nanocomposites.^83^ CNTs allow ease surface chemical modification either in the form of chemical bonding or van der Waals force to form a nanocarbon hybrid.^84^ The first demonstration of Z‐schematic water splitting system using CNTs was reported by Ng et al. in which Zn_0.5_Cd_0.5_S and TiO_2_ were employed as PS I and II, respectively.[qv: 17a] The ternary Zn_0.5_Cd_0.5_S‐CNTs‐TiO_2_ nanocomposites synthesized via two‐step facile coating and hydrothermal route demonstrated ≈4.5‐ and 2.8‐fold increment in photocatalytic H_2_ evolution over Zn_0.5_Cd_0.5_S and CNTs‐Zn_0.5_Cd_0.5_S, respectively. As delineated in **Figure** **27**, the ternary nanocomposites display an intimate interfacial interaction between the three components and confer a plausible Z‐schematic cascade electron transfer. The unprecedented properties and highly tailoring nature of CNTs open up new opportunities for the next generation of photocatalytic system, particularly to the Z‐scheme water splitting.

**Figure 28:**
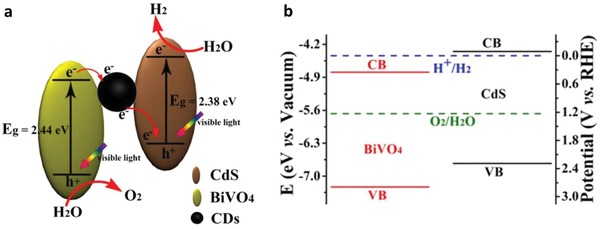
Schematic illustration of a) charge transfer mechanism and b) its corresponding band position of BiVO_4_‐CQDs‐CdS Z‐scheme system. Adapted with permission.^85^ Copyright 2017, Elsevier.

CQDs are amenable to function as electron mediator in PS‐C‐PS system attributed to their excellent charge relaying properties. A ternary system which comprises of BiVO_4_‐CQDs‐CdS was developed by Wu et al. for photocatalytic overall water splitting under visible light, achieving a stoichiometric evolution of H_2_ (1.24 µmol h^−1^) and O_2_ (0.61 µmol h^−1^) when the mass ratio of PS I to II was 1:1.^85^ As displayed in **Figure** **28**, the as‐synthesized ternary nanocomposites postulate plausible vectorial electron transfer from BiVO_4_ (CB) to CdS (VB) through CQDs as the electron shuttling channel. As a result, this system demonstrates suitable band position for overall water splitting with strong reduction and oxidation abilities. In another similar study, a thin carbon layer was employed to interface 2D g‐C_3_N_4_ nanosheets and ZnInS_4_ (ZIS) to form a ternary nanocomposite with Z‐scheme electronic configuration.^86^ The resultant sample is able to perform photocatalytic H_2_ evolution up to 50.32 µmol h^−1^ under Na_2_S/Na_2_SO_3_ sacrificial condition. As is known, pristine g‐C_3_N_4_ commonly demonstrates sluggish H_2_ evolution in the absence of co‐catalysts, Pt. However, the incorporation of g‐C_3_N_4_ into the Z‐scheme system is able to render high H_2_ evolution even without Pt, which implies the advantages of facilitated charge transfer and bestow suitable dynamics for photocatalytic reaction.

Attributed to the competency of various metals and nanocarbons in driving electron transfer, PS‐C‐PS system has been highlighted as an alternative to govern Z‐scheme photocatalysis than its forebear PS‐A/D‐PS system. Detrimental backward reaction and shielding effect can be perfectly evited due to the absence of redox ionic pairs. Besides, nanocarbon‐based mediator is a relatively new addition to the family of PS‐C‐PS system. Albeit still not prevalent as the employment of metallic mediator, there is a growing interest into the exploration of nanocarbon materials such as graphene, CNTs and CQDs in driving Z‐scheme system. Despite promising results on both sacrificial aided and pure water splitting for H_2_ production were shown by nanocarbon‐mediated Z‐scheme system, extra attention should be devoted to the synthesis of such nanocomposites. An intimate contact interface between photocatalysts and mediator is of paramount significance to provide a low‐resistance pathway for continuous vectorial electron flow from PS II to PS I. Besides, the electron relaying ability of nanocarbon is commonly plagued by the formation of defects during the surface modification. The extent of these defects could be diminished via post reduction of oxygenated graphitic structure. However, excessive reduction of oxygenated groups will turn the nanocarbon materials into hydrophobic behavior, which make their dispersion in water harder. Nevertheless, the intriguing properties of all‐solid‐state Z‐scheme system have raised considerable attention worldwide to develop highly efficient H_2_ production devices that resemble natural photosynthesis. **Table** **7** depicts the summary of PS‐C‐PS system that has been developed for water splitting.

**Table 7:** Summary of PS‐C‐PS system for water splitting

Entry	PS I (available λ)	PS II (available λ)	Mediator	Light source	Solution	Efficiency	Ref.
1	Pt/CdS (<540 nm)	TiO_2_ (<387 nm)	Au	500 W Xe lamp (300 < λ < 400 nm)	Pure water (20 mL)	H_2_: 10 µmol h^−1^	23
2	Pt/CdS (<540 nm)	TiO_1.96_C_0.04_ (<455 nm)	Au	300 W Xe lamp (λ > 420 nm)	0.05 M Na_2_S/0.1 m Na_2_SO_3_ (120 mL)	H_2_: 433.2 µmol h^−1^; AQY: 23.6% (420 nm)	68
3	CdS (<540 nm)	ZnO (<387 nm)	Au	300 W Xe lamp	0.1 m Na_2_S/0.1 m Na_2_SO_3_ (270 mL)	H_2_: 60.8 µmol h^−1^	[qv: 67a]
4	CdS (<540 nm)	WA‐TiO_2_ (<387 nm)	Au	750 W Xe lamp	0.25 m Na_2_S/0.35 m Na_2_SO_3_ (50 mL)	H_2_: 3.2 µmol h^−1^	69
5	ZnRh_2_O_4_ (<1033 nm)	Ag_1−_ *_x_*SbO_3−_ *_y_* (<460 nm)	Ag	300 W Xe lamp (λ > 460 nm)	Pure water	AQY: 0.04% (420 nm)	87
6	Ag_2_S (<1239 nm)	TiO_2_ (<387 nm)	Ag	300 W Xe lamp	0.25 m Na_2_S/0.35 m Na_2_SO_3_ (25 mL)	H_2_: 0.63 µmol h^−1^	70
7	PbBi_2_Nb_1.9_Ti_0.1_O_9_ (<430 nm)	WO_3_ (<460 nm)	W	450 W Xe lamp (λ > 420 nm)	15 vol% methanol	H_2_: 14.8 µmol h^−1^	88
8	Pt/CdS (<540 nm)	ZnO (<387 nm)	Cd	300 W Xe lamp	0.1 m Na_2_S/0.1 m Na_2_SO_3_ (300 mL)	H_2_: 1.92 mmol h^−1^	66
9	Ru/SrTiO_3_:La,Rh (<500 nm)	CoO*_x_*/Ta_3_N_5_ (<600 nm)	Ir	300 W Xe lamp (λ > 420 nm)	DI water adjusted by H_2_SO_4_ (pH = 3.9)	AQY: 1.1% (420 nm)	71
10	Ru/SrTiO_3_:Rh (<520 nm)	BiVO_4_ (<520 nm)	RGO	300 W Xe lamp (λ > 420 nm)	DI water adjusted by H_2_SO_4_ (pH = 3.5)	H_2_: 11 µmol h^−1^; O_2_: 5.5 µmol h^−1^	25
11	Pt/CaGaS_2_ (<520 nm)	TiO_2_ (<387 nm)	RGO	300 W Xe lamp	Pure water (120 mL)	H_2_: 19.8 µmol h^−1^; O_2_: 10.3 µmol h^−1^	77
12	CdS (<540 nm)	ZnO (<387 nm)	RGO	300 W Xe lamp	0.1 m Na_2_S/0.1 m Na_2_SO_3_ (300 mL)	H_2_: 0.51 mmol h^−1^	89
13	N‐RGO (<564 nm)	O‐RGO (<564 nm)	RGO	300 W Xe lamp (420 < λ < 800 nm)	Pure water (200 mL)	H_2_: 1.2 µmol h^−1^; O_2_: 0.7 µmol h^−1^	80
14	Pt/CaGaS_2_ (<520 nm)	CoO*_x_*/BiVO_4_ (<520 nm)	RGO	300 W Xe lamp (λ > 420 nm)	Pure water (120 mL)	H_2_: 3.5 µmol h^−1^; O_2_: 1.7 µmol h^−1^	78
15	g‐C_3_N_4_ (<424 nm)	Cd_0.5_Zn_0.5_S (<514 nm)	RGO	300 W Xe lamp	0.35 m Na_2_S/0.25 m Na_2_SO_3_ (100 mL)	H_2_: 1.18 mmol h^−1^	90
16	Pt/PCN (<443 nm)	Fe_2_O_3_ (<600 nm)	RGO	300 W Xe lamp (λ > 400 nm)	Pure water (100 mL)	H_2_: 43.6 µmol h^−1^; O_2_: 21.2 µmol h^−1^	91
17	ZnIn_2_S_4_ (<575 nm)	g‐C_3_N_4_ (<460 nm)	C	3 W UV‐LEDs	0.5 m Na_2_S/0.5 m Na_2_SO_3_ (80 mL)	H_2_: 50.32 µmol h^−1^	86
18	CdS (<540 nm)	BiVO_4_ (<520 nm)	CQDs	300 W Xe lamp (λ > 420 nm)	Pure water (100 mL)	H_2_: 1.24 µmol h^−1^; O_2_: 0.61 µmol h^−1^	85
19	Zn_0.5_Cd_0.5_S (<540 nm)	TiO_2_ (<400 nm)	CNTs	500 W Xe lamp (AM 1.5)	0.1 m Na_2_S/0.1 m Na_2_SO_3_ (120 mL)	H_2_: 21.9 µmol h^−1^	[qv: 17a]

### 3.3 PS‐PS System or Direct Z‐Scheme (Third Generation)

#### 3.3.1 Mechanism of Electron Mediator

**Figure 29:**
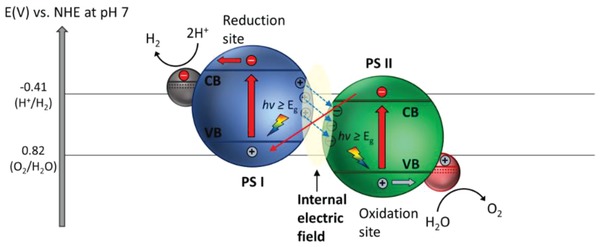
Schematic band energy diagram of PS‐PS or direct Z‐scheme system.

Apart from the compelling electron transfer ability displayed by metallic and nanocarbon‐based materials as conductor, solid–solid contact interface also renders similar energy profile which is quasi‐continuous.^24^ The aggregated defects on the contact interface between two semiconductors could potentially induce an internal electric field that bestows low electric resistance for charge transfer, known as Ohmic contact.^92^ In other words, this solid–solid contact interface could potentially be utilized to perform Z‐schematic vectorial electron transfer between semiconductors. As shown in **Figure** **29**, the delocalized electrons after light illumination in PS II (CB) will be shuttled to PS I (VB) ascribed to the strong electrostatic attraction between opposite charge clouds. In this regard, the electron transfer from PS I (CB) to PS II (CB) will be negligible due to the strong electrostatic repulsion, which omits the potential of type‐II heterojunction formation. Hence, the photoexcited electrons and holes will be accommodated at the highest possible CB and lowest possible VB of this Z‐scheme system to govern a stronger redox ability as compared to conventional type‐II heterojunction. This PS‐PS system without an external mediator, also known as direct Z‐scheme, marks the new generation of two‐step photoexcitation system. One of the key factors for the formation of direct Z‐scheme is the properties of solid–solid contact interface between PS I and II. Thus, the formation method of this interface is of utmost important in order to determine the degree of resistance for charge transfer.^21^ In this context, the electrical resistance can be adjusted by modification of semiconductors during the construction of solid–solid interface.

**Figure 30:**
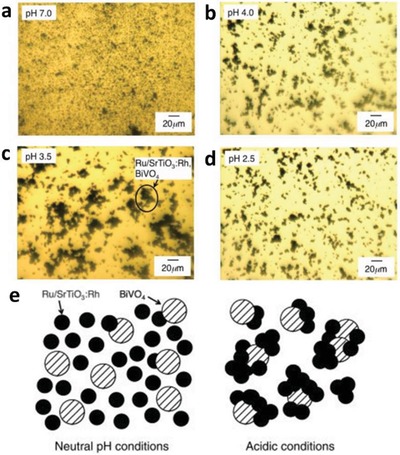
Physical formation of direct Z‐scheme. Optical microscope images of aqueous suspension containing Ru/SrTiO_3_:Rh and BiVO_4_ under different pH. a) 7, b) 4, c) 3.4, and d) 2.5. e) Schematic illustration of contact behaviors of Ru/SrTiO_3_:Rh‐BiVO_4_ suspension under neutral and acidic conditions. Adapted with permission.^27^ Copyright 2009, American Chemical Society.

The formation of solid–solid contact in direct Z‐scheme is generally divided into two main categories, namely physical and chemical methods. Physical contact mode of direct Z‐scheme can be realized when the two photosystems possess opposite charges in an aqueous medium, and hence, the strong attraction will induce an interparticle electrostatic adsorption to joint PS I and II. The first demonstration of PS‐PS system formed by physical means was witnessed in 2009 by Sasaki et al., in which Ru/SrTiO_3_:Rh‐BiVO_4_ composite was developed in aqueous suspension at pH 3.5.^27^ It has been well entrenched that the surface charge of semiconductor is highly dependent on its isoelectric point and the pH of solution. As depicted in **Figure** **30**, the suspension shows the most aggregated behavior of Ru/SrTiO_3_:Rh‐BiVO_4_ at pH 3.5 in contrast to other pH values (7, 4, and 2.5). This reveals that the strongest physical interaction between Ru/SrTiO_3_:Rh and BiVO_4_ happens at this pH, which is further corroborated by their high activity in photocatalytic water splitting with measured AQY of 1.7% at 420 nm.^27^ This observation was further validated by the examination of zeta potential of respective photosystems under different pH. The result shows that at pH 3.5, both Ru/SrTiO_3_:Rh and BiVO_4_ simultaneously exhibited opposite charges. The zeta potential of BiVO_4_ is negative across a wide range of pH from 2 to 9, whereas Ru/SrTiO_3_:Rh confers isoelectric point of ≈4. As a result, the opposite charges of both Ru/SrTiO_3_:Rh and BiVO_4_ at pH 3.5 deliver a strong interaction which leads to the formation of solid–solid contact, as delineated in Figure 30e.

**Figure 31:**
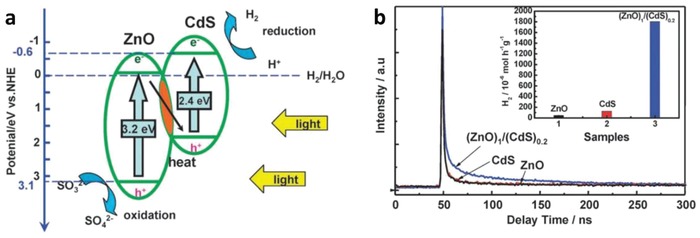
Chemical formation of direct Z‐scheme. a) Schematic illustration of band structure of ZnO/CdS direct Z‐scheme. b) Fluorescence emission decay spectra of ZnO, CdS and (ZnO)_1_/(CdS)_0.2_. Adapted with permission.[qv: 26a] Copyright 2009, Royal Society of Chemistry.

Contrary to the physical construction method, chemical contact mode of PS‐PS system emphasizes on the chemical bond formation between PS I and II. In typical, one of the photosystems will be firstly synthesized and it serves as the potent platform for the subsequent loading of another photosystem. Consequently, the conjugation of surface will confer strong chemical bond, which is more stable than the surface interaction formed by physical mean. For instance, ZnO/CdS system was developed using wet chemistry method which displayed Z‐schematic cascade electron profile as shown in **Figure** **31**a.[qv: 26a] ZnO was initially synthesized through the precipitation of Zn(OH)_2_ from its precursor Zn(CH_3_COO)_2_.2H_2_O when the pH of the suspension was adjusted to 7 using aqueous ammonia. Subsequently, CdS was formed on top of ZnO as a facile template to outline a two‐component system. Attributed to the unique vectorial transfer between ZnO and CdS, the two‐component system renders a prolonged lifetime of photogenerated carriers as compared to the single‐component ZnO and CdS. The charge transport properties can be elucidated from the decay lifetime of charge dynamics shown in fluorescence emission spectra (Figure 31b). In addition, the Z‐scheme heterostructures demonstrated higher photocatalytic activity as compared to single‐component system, leading to H_2_ evolution rate of 774 µmol h^−1^ under sacrificial condition (0.1 m Na_2_S/Na_2_SO_3_). In all, the facilitated charge transport and enhanced photocatalytic performance in PS‐PS system are credited to the formation of low resistance interface between solid–solid contact. This substantiates the feasibility of direct Z‐scheme in achieving two‐step photoexcitation without any mediator. Verification of Z‐scheme electron transfer profile in PS‐PS system will be discussed henceforth.

#### 3.3.2 Verification of Direct Z‐Scheme

As aforementioned, the band structures of both direct Z‐scheme and type‐II heterojunction are of utmost similar due to the arrangement of semiconductors. However, both systems confer totally reverse electron flow profiles ascribed to the properties of solid–solid interfaces. Thus, it is imperative to investigate the charge transfer mechanism of the direct Z‐scheme system via different testing and characterization in order to differentiate them from the conventional heterojunction‐typed nanocomposites. Up to now, various methods have been employed to study and verify the electronic structure of direct Z‐scheme, as outlined in the following sections. This includes metal loading, sacrificial reagent testing, radical trapping experiment, and X‐ray photoelectron spectroscopy (XPS) testing for the validation of direct Z‐scheme. However, a single experiment is out of precision to corroborate the vectorial charge transfer profile in direct Z‐scheme and hence, a series of comprehensive testing should be conducted to confirm its electronic structure.

##### Metal Loading

**Figure 32:**
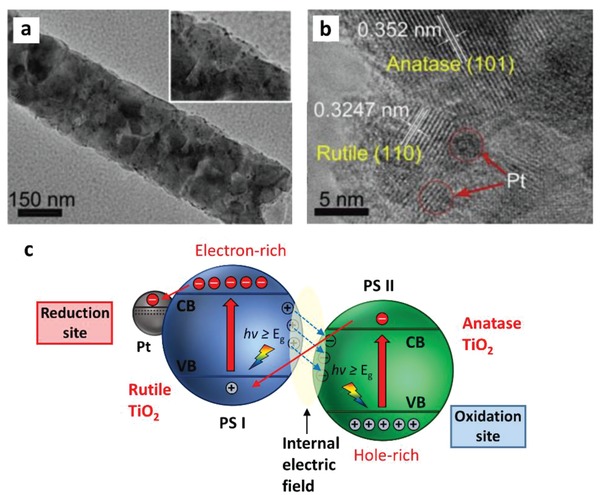
Metal loading method for direct Z‐scheme verification. a,b) TEM and HRTEM images of anatase/rutile TiO_2_ nanocomposites loaded with Pt nanoparticles. c) Plausible electronic profile of anatase/rutile TiO_2_ nanocomposites during photodeposition of Pt. Adapted and reproduced with permission.^93^ Copyright 2014, Elsevier.

Photodeposition of noble metal as co‐catalysts is one of the typical methods to verify the charge transfer profile in direct Z‐scheme. As opposed to type‐II heterojunction, the interaction in solid–solid contact within direct Z‐scheme causes the redistribution of charge carriers ascribed to the vectorial electron transfer via Ohmic contact from PS II (CB) to PS I (VB). Thus, PS I is an electron‐rich region while PS II is aggregated with holes, as displayed in **Figure** **32**c. In this context, metal will exist in its cationic form in the solution containing its corresponding precursors. Hence, the positively charged cation will be attracted toward the electron‐rich side of PS I under irradiation of light. As a result, PS I will be photodeposited with metal nanoparticles as co‐catalysts, providing the charge transfer profile within the system which follows a Z‐scheme configuration. Xu et al. implemented this method in testifying the direct Z‐scheme mechanism of anatase/rutile TiO_2_ nanocomposites.^93^ As depicted in Figure 32a,b, Pt nanoparticles were found to be photodeposited on rutile TiO_2_ which serves as PS I for the direct Z‐scheme system. Thus, the accumulation of photogenerated electrons on PS I is validated, which indicates the migration of electrons from anatase to rutile TiO_2_.

##### Sacrificial Reagent Testing

**Figure 33:**
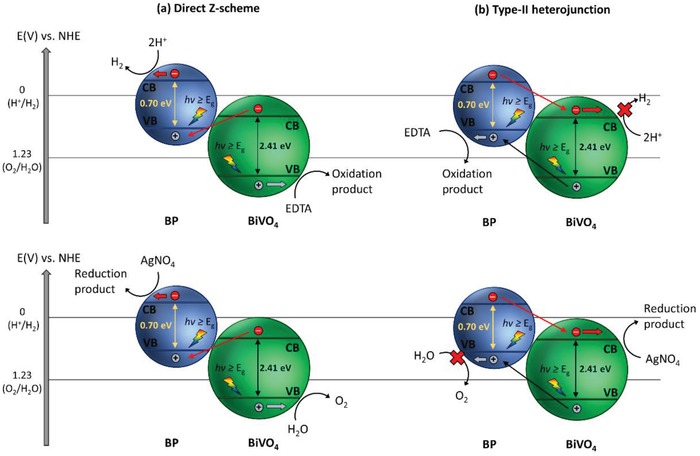
Charge transfer mechanism in direct Z‐scheme and heterojunction. a) Direct Z‐scheme and b) type‐II heterojunction of BP‐BiVO_4_ coupling during sacrificial testing. Reproduced with permission.^94^ Copyright 2018, Wiley.

Similar to metal loading experiment, the employment of sacrificial reagent testing can determine the region of aggregated electrons and holes within the Z‐scheme system. In order to be validated as direct Z‐scheme, the two‐component system will have to fulfill three conditions: 1) PS I can only perform H_2_ evolution under sacrificial electron donors, 2) PS II can only perform O_2_ evolution under sacrificial electron acceptors, and 3) the two‐component PS I‐PS II system is able to perform overall water splitting in pure water, leading to simultaneous evolution of H_2_ and O_2_. In a more recent work, Zhu et al. performed sacrificial reagent testing to verify the cascade electron transfer profile within black phosphorus (BP)‐BiVO_4_ nanocomposites.^94^ As tabulated in **Table** **8**, both Co_3_O_4_‐loaded BP and BiVO_4_ can render H_2_ and O_2_ activities under EDTA and AgNO_4_, respectively (entries 1–8). This proves the capabilities of individual PS I and II to perform their corresponding half reaction via reduction and oxidation reaction. The coupling of Co_3_O_4_/BP‐Co_3_O_4_/BiVO_4_ was tested under the aforementioned three conditions: 1) EDTA electron donors, 2) AgNO_4_ electron acceptors, and 3) pure water. As shown from entry 9, the two‐component system was able to demonstrate photocatalytic H_2_ evolution under EDTA without any O_2_ being detected. Similarly, Co_3_O_4_/BP‐Co_3_O_4_/BiVO_4_ shows O_2_ evolution under AgNO_4_ with no H_2_ product, which consolidates the electron cloud was isolated at BP while BiVO_4_ was aggregated with photogenerated holes, as delineated in **Figure** **33**. Moreover, the photocatalytic water splitting reaction was further tested under pure water without any sacrificial reagent, resulting in simultaneous evolution of H_2_ and O_2_ gas with close to stoichiometric value (entry 12). Thus, sacrificial reagent testing is feasible to be utilized for determining the plausible charge transfer mechanism in direct Z‐scheme. However, this application is only suitable for coupling of semiconductors with band structure that is solely responsible for H_2_ or O_2_ evolution. The electron transfer within a semiconductor with bandgap that can fulfill both H_2_ and O_2_ activities will be hard to be determined since it can respond to both electron donor and acceptors in the half reaction testing.

**Table 8:** Sacrificial reagent testing for direct Z‐scheme verification. Water splitting activities of different samples with and without sacrificial reagent under visible light. Adapted with permission.^94^ Copyright 2018, Wiley

Entry	PS I	PS II	Sacrificial reagent	Activities [µmol h^−1^]	
				H_2_	O_2_
1	BP	–	–	0	0
2	BP	–	EDTA	0.09	0
3	Co_3_O_4_/BP	–	–	0.59	0
4	Co_3_O_4_/BP	–	EDTA	2.1	0
5	–	BiVO_4_	–	0	0.13
6	–	BiVO_4_	AgNO_4_	0	0.65
7	–	Co_3_O_4_/BiVO_4_	–	0	0.37
8	–	Co_3_O_4_/BiVO_4_	AgNO_4_	0	2.2
9	Co_3_O_4_/BP	Co_3_O_4_/BiVO_4_	EDTA	7.5	0
10	Co_3_O_4_/BP	Co_3_O_4_/BiVO_4_	AgNO_4_	0	4.2
11	BP	BiVO_4_	–	0.8	0.51
12	Co_3_O_4_/BP	Co_3_O_4_/BiVO_4_	–	3.9	2.3

##### Radical Trapping Experiment

**Figure 34:**
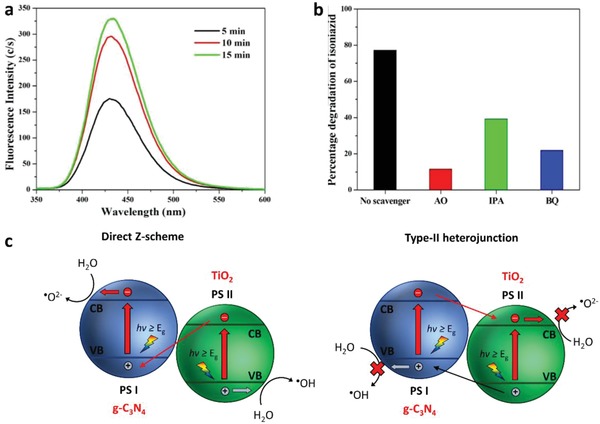
Radical testing for direct Z‐scheme verification. a) PL spectra of g‐C_3_N_4_/TiO_2_ nanocomposites in solution containing 5 × 10^−4^ M TA under irradiation (excitation at 315 nm). b) Effect of scavengers on degradation of ISN using g‐C_3_N_4_/TiO_2_ nanocomposites. c) Charge transfer mechanism of g‐C_3_N_4_/TiO_2_ in both direct Z‐scheme and type‐II heterojunction systems. Adapted and reproduced with permission.^97^ Copyright 2015, Elsevier.

According to kinetic theory of heterogeneous photocatalysis, hydroxyl radicals (^•^OH) will be produced from oxidation of water when the semiconductor possesses more electropositive potential than 2.4 versus NHE.^95^ On the other hand, superoxide radicals (^•^O^2−^) are the reduction products from water if the semiconductor fulfills sufficient potential of −0.33 V versus NHE.^96^ With the thermodynamics constraint placed for the generation of these radicals, it is possible to differentiate direct Z‐scheme from conventional type‐II heterojunction with radical trapping experiment since the photogenerated carriers are accommodated at different potential levels among the two systems. In a study by Jo et al. to substantiate the direct Z‐scheme configuration of g‐C_3_N_4_/TiO_2_ nanocomposites, radical testing of ^•^OH and ^•^O^2−^ was investigated using terephthalic acid (TA) and 1,4‐benzoquinone (BQ) as probe molecules.^97^ As is known, ^•^OH radicals are readily to react with TA and form a fluorescence product, namely, 2‐hydroxyterephthalic acid. These products can be detected using photoluminescence (PL) measurements, in which a corresponding peak will be observed at wavelength ≈430 nm.^98^ **Figure** **34**a shows that the PL intensity increases over time for the photocatalytic reaction, indicating the generation of ^•^OH radical from g‐C_3_N_4_/TiO_2_ nanocomposites. This finding elucidates the strong oxidation ability of the two‐component system imputed to the isolation of holes at VB of TiO_2_. Besides, several scavengers were used to quench the presence of radicals during degradation of isoniazid (ISN), which includes isopropyl alcohol (IPA), ammonium oxalate (AO) and BQ associated to scavenge ^•^OH, holes and ^•^O^2^. As shown in Figure 34b, the percentage of degradation was substantially increased upon the addition of scavengers individually. Hence, the presence of ^•^OH and ^•^O^2^ as main radicals during photocatalytic reaction of g‐C_3_N_4_/TiO_2_ nanocomposites can be confirmed. This gives a solid proof on the plausible accumulation of photogenerated electrons and holes at g‐C_3_N_4_ and TiO_2_ separately, as depicted in Figure 34c.

##### XPS Testing

**Figure 35:**
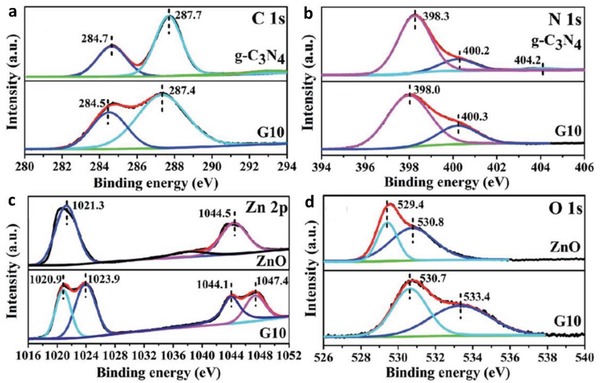
XPS method for direct Z‐scheme verification. High resolution XPS spectra of a) C 1s and b) N 1s of g‐C_3_N_4_ and g‐C_3_N_4_/ZnO. High resolution XPS spectra of c) Zn 2p and d) O 1s of ZnO and g‐C_3_N_4_/ZnO. Adapted with permission.^99^ Copyright 2015, Royal Society of Chemistry.

**Figure 36:**
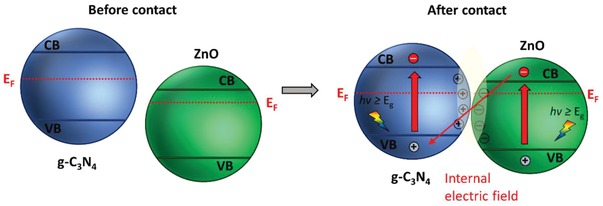
Formation of direct Z‐scheme. Schematic illustration of electronic band structure diagram of g‐C_3_N_4_/ZnO before and after contact. Reproduced with permission.^22^ Copyright 2017, Wiley.

XPS analysis has been widely employed to determine the chemical composition and surface states of materials. As of late, the application of XPS has been extended to gain further insights on the changes in electron density via examination in the shifting of binding energies of constituent elements with reference to C 1s as the calibration peak. In an investigation on the direct Z‐scheme mechanism of g‐C_3_N_4_/ZnO by Yu et al., the deconvoluted N 1s XPS peak of g‐C_3_N_4_/ZnO displays an obvious peak shift toward lower energy region as compared to g‐C_3_N_4_ (**Figure** **35**b), whereas O 1s XPS peak of g‐C_3_N_4_/ZnO shifted toward higher energy region in contrast to ZnO.^99^ This phenomena can be well explained by the change in electron density. A positive shift in binding energy of XPS peak indicates a decrease in electron density, whereas a negative shift reflects an increase in the electron cloud. Thus, the pathway of electron transfer within g‐C_3_N_4_/ZnO is predicted to be dictated from ZnO to g‐C_3_N_4_, ascribed to the decrease in electron density of ZnO associated to the increase in binding energy of O 1s XPS spectra. Furthermore, the electron migration pathway can be justified by the formation of an internal electric field on the solid–solid contact. As shown in **Figure** **36**, the coupling of g‐C_3_N_4_ and ZnO induces the rearrangement of band structure due to the difference in Fermi level of each semiconductor. Since g‐C_3_N_4_ possesses a higher Fermi level, electrons tend to migrate from g‐C_3_N_4_ to ZnO during the Fermi level alignment, causing g‐C_3_N_4_ to be positively charged. On the other hand, ZnO received electrons from g‐C_3_N_4_ and became negatively charged. Therefore, the solid–solid interface was charged and it prompts the generation of weak internal electric field. Thus, photogenerated electron from ZnO (CB) will preferably transport to g‐C_3_N_4_ (VB) via the low resistance interface, accomplishing the Z‐schematic electronic configuration. Hence, XPS analysis is an efficient tool to differentiate direct Z‐scheme from type‐II heterojunction.

#### 3.3.3 Application in Photocatalytic Water Splitting

**Figure 37:**
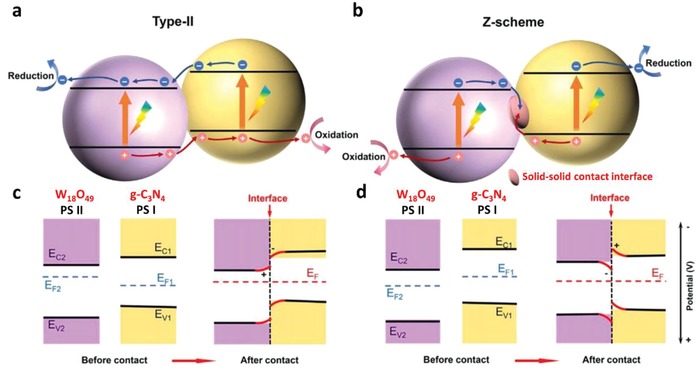
Heterojunction and direct Z‐scheme in g‐C_3_N_4_/W_18_O_49_. a,b) Electronic band structure diagram and c,d) energy diagram during Fermi level alignment in type‐II heterojunction and Z‐scheme system. Adapted and reproduced with permission.^100^ Copyright 2017, Elsevier.

**Figure 38:**
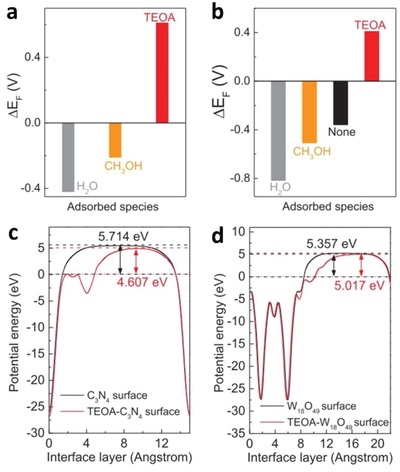
Fermi level difference between g‐C_3_N_4_ and W_18_O_49_ with and without addition of sacrificial reagent measured by a) OCP and b) DFT. Calculated potential measured by DFT of c) g‐C_3_N_4_ and d) W_18_O_49_. Adapted with permission.^100^ Copyright 2017, Elsevier.

In view of the exponential growing in popularity of graphitic carbon nitride (g‐C_3_N_4_), there is a huge interest into the exploration of g‐C_3_N_4_ based Z‐scheme system in water splitting. As stated, the successful coupling of direct Z‐scheme relies on the formation of internal electric field within the solid–solid contact interface. In other words, the Fermi levels of the two photocatalysts that are about to be coupled will play a significant role on the final electronic configuration. A recent intrinsic insight on the interfacial properties of g‐C_3_N_4_/W_18_O_49_ nanocomposites was found to render switchable charge transfer behaviors via the tuning of band bending.^100^ As shown in **Figure** **37**c, g‐C_3_N_4_ has more electronegative band structure which is suitable to serve as PS I while W_18_O_49_ with a lower band configuration can bestow strong oxidation ability as PS II. However, the Fermi level of g‐C_3_N_4_ is lower than W_18_O_49_ which induces electron injection from W_18_O_49_ to g‐C_3_N_4_ during the Fermi level alignment. As a result, contact interface of g‐C_3_N_4_ will be negatively charged, whereas W_18_O_49_ will be positively charged. In this regard, the energy bands of g‐C_3_N_4_ will be bending downward in contrary to the upward band bending of W_18_O_49_. Thus, g‐C_3_N_4_/W_18_O_49_ nanocomposites confer type‐II heterojunction electronic configuration in normal circumstances. Due to the charge isolation profile of type‐II heterojunction shown in Figure 37a, the reduction and oxidation potentials are greatly weakened. Surprisingly, with the introduction of TEOA as additives, the Fermi level of g‐C_3_N_4_ was upshifted and accommodated in a more electronegative position than that of W_18_O_49_. The calculated work function (WF) of g‐C_3_N_4_ was found to have decreased from 5.714 to 4.607 eV, which implies the upshifting of Fermi level after the addition of TEOA (**Figure** **38**c). On the other hand, the WF of W_18_O_49_ was slightly affected with variation from 5.357 to 5.017 eV from the DFT calculation in Figure 38d. Subsequently, the Fermi level difference between g‐C_3_N_4_ and W_18_O_49_ was estimated according to the calculated WF, revealing the uplifting of Fermi level of g‐C_3_N_4_ to be more electronegative than W_18_O_49_ after the adsorption of TEOA (Figure 38a,b). Therefore, a new electronic configuration is conferred by the g‐C_3_N_4_/W_18_O_49_ nanocomposites after the addition of TEOA as shown in Figure 38d. With the adjustment of Fermi level after contact, the internal electric field is generated at the contact interface with band of g‐C_3_N_4_ bending upward. Hence, the photogenerated electrons and holes will be isolated at the highest possible CB and lowest possible VB to govern strong water splitting reaction. The visible‐light‐driven photocatalytic water splitting activity of g‐C_3_N_4_/W_18_O_49_ nanocomposites was tested under TEOA condition, which yielded a H_2_ evolution rate of 51.02 µmol h^−1^.^100^

**Figure 39:**
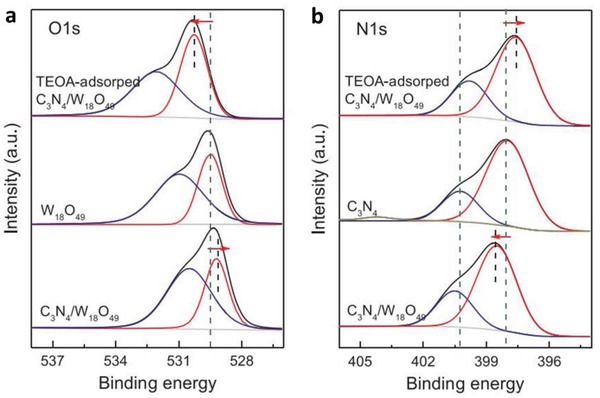
High resolution XPS of TEOA‐adsorbed g‐C_3_N_4_/W_18_O_49_, pure g‐C_3_N_4_/W_18_O_49_ and their respective individual components of a) O 1s and b) N 1s. Adapted with permission.^100^ Copyright 2017, Elsevier.

Additionally, XPS was employed by Huang et al. to determine the migration of charge carrier in g‐C_3_N_4_/W_18_O_49_ nanocomposites.^100^ As displayed in **Figure** **39**, it can be observed that O 1s shifts toward higher binding energy while N 1s dictates toward lower binding energy when comparing TEOA‐adsorbed g‐C_3_N_4_/W_18_O_49_ nanocomposites with the pristine counterpart and their individual components (g‐C_3_N_4_ and W_18_O_49_). This elucidates the transfer of electrons occur from W_18_O_49_ to g‐C_3_N_4_ ascribed to the increase in electron density of g‐C_3_N_4_, accomplishing a cascade electron transfer within the system that resembles direct Z‐scheme. In whole, it is important to modulate the Fermi level of semiconductors to form a direct Z‐scheme nanocomposites rather than a type‐II heterojunction in order to govern a considerable large overpotential for highly efficient water splitting reaction.

**Figure 40:**
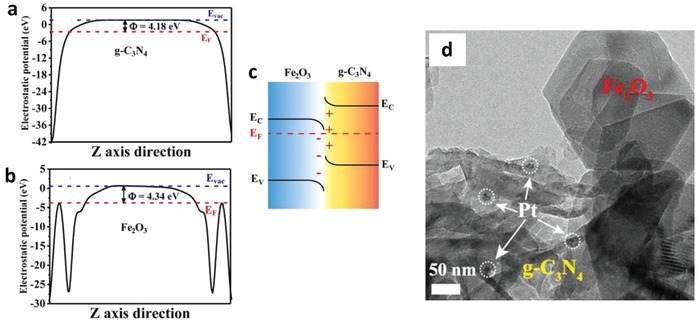
Electronic configuration of direct Z‐scheme in g‐C_3_N_4_/Fe_2_O_3_. Calculated WF of a) g‐C_3_N_4_ and b) Fe_2_O_3_. c) Estimated band configuration of g‐C_3_N_4_/Fe_2_O_3_. d) HRTEM image of Pt‐loaded g‐C_3_N_4_/Fe_2_O_3_. Adapted with permission.^101^ Copyright 2018, Wiley.

**Figure 41:**
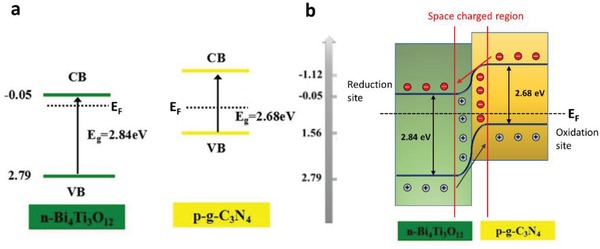
Electronic configuration of p–n junction in g‐C_3_N_4_/Bi_4_Ti_3_O_12_. a) Band structure diagram of g‐C_3_N_4_ and Bi_4_Ti_3_O_12_. b) Charge transfer mechanism of g‐C_3_N_4_/Bi_4_Ti_3_O_12_ nanocomposites. Adapted and reproduced with permission.^102^ Copyright 2016, Elsevier.

Similarly, Xu et al. utilized the conjugation between Fe_2_O_3_ and g‐C_3_N_4_ in assembling a 2D/2D structured direct Z‐scheme system.^101^ As delineated in **Figure** **40**d, intimate contact interface between Fe_2_O_3_ and g‐C_3_N_4_ with uniform deposition of Pt can be clearly visualized. The intrinsic insight on the contact interface was calculated using DFT, which revealed g‐C_3_N_4_ has a lower WF and a higher Fermi level than Fe_2_O_3_. Consequently, a band configuration of direct Z‐scheme is formed within the nanocomposites, in which photogenerated electron was accumulated in g‐C_3_N_4_ (CB) and holes being isolated in Fe_2_O_3_ (VB). Besides, g‐C_3_N_4_/Fe_2_O_3_ exhibited photocatalytic H_2_ evolution rate of 19.9 µmol h^−1^ under TEOA, which is ≈13‐fold enhancement over pristine g‐C_3_N_4_. Interestingly, the formation of p–n interface can induce similar space charged region as the internal electric field of direct Z‐scheme. In a study on the coupling of g‐C_3_N_4_ and Bi_4_Ti_3_O_12_, a type‐II heterojunction with p–n interface was formed ascribed to the p‐type semiconductor nature of g‐C_3_N_4_ and n‐type of Bi_4_Ti_3_O_12_.^102^ Different from the direct Z‐scheme of the aforesaid g‐C_3_N_4_/Fe_2_O_3_, the formation of space charged region within g‐C_3_N_4_/Bi_4_Ti_3_O_12_ is attributed to the diffusion of electron across p–n interface, as depicted in **Figure** **41**. In an effort to differentiate the effect of space charged region between the solid–solid interface of direct Z‐scheme and p–n heterojunction, it can be observed that the Fermi level of PS I (g‐C_3_N_4_) is lower than PS II (Bi_4_Ti_3_O_12_). As a result, the interface was negatively charged at PS I while positive at PS II. Hence, electron will be driven from PS I (CB) to PS II (CB). Whereas for direct Z‐scheme, Fermi level of PS I is higher than PS II, which results in the formation of positively charged interface at PS I and negative at PS II. Thus, cascade electron transfer profile can be witnessed in direct Z‐scheme system. Even though the presence of internal electric field in p–n junction nanocomposites can facilitate electron–hole pairs separation, redox ability of the system can be greatly reduced. In all, the formation of direct Z‐scheme can be realized when the Fermi level of PS I is higher than PS II as it provides synergy in band alignment for vectorial electron flow.

In conclusion, PS‐PS system offers a new dimension for the construction of a two‐step photoexcitation system without any electron mediator. The summary of PS‐PS system or direct Z‐scheme is presented in **Table** **9**. The cost of Z‐scheme system can be greatly reduced when omitting the needs of metallic or nanocarbon‐based conductor. However, PS‐C‐PS system generally shows better charge transport ability with the incorporation of conductor as the electron mediator. This is ascribed to the difference in electrical resistance between metallic/nanocarbon mediator and semiconductor, which facilitates the charge transport process.^24^ As aforesaid, the quasi‐continuous energy state at the solid–solid interface of direct Z‐scheme can confer internal electric resistance that is favorable for driving low resistance electron transfer. Similar to the space charged region of p–n heterojunction, this internal electric field is formed via the Fermi level alignment of two semiconductors with different WF. Even so, direct Z‐scheme can only be formed when PS I possesses a higher Fermi level than PS II, leading to a positively charged interface of PS I. Hence, electron will be dictated from negative charged PS II to PS I, accomplishing vectorial charge transfer. Thus, coupling of two semiconductors with lower Fermi level of PS I will induce the formation of type‐II or p–n heterojunction. Besides, it is necessary to undergo several verification testing, i.e., metal loading, sacrificial reagent testing, radical trapping experiment, and XPS method to differentiate direct Z‐scheme from type‐II heterojunction‐typed system.

**Table 9:** Summary of PS‐PS system for water splitting

Entry	PS I (available λ)	PS II (available λ)	Light source	Solution	Efficiency	Ref.
1	Ru/SrTiO_3_:Rh (<520 nm)	BiVO_4_ (<540 nm)	300 W Xe lamp (λ > 420 nm)	DI water adjusted by H_2_SO_4_ (pH = 3.5; 120 mL)	AQY: 1.7% (420 nm)	27
2	Pt/CdS (<540 nm)	ZnO (<387 nm)	300 W Xe lamp	0.1 m Na_2_S/0.1 m Na_2_SO_3_ (300 mL)	H_2_: 774 µmol h^−1^	[qv: 26a]
3	Ru/SrTiO_3_:Rh (<520 nm)	Ir/CoO*_x_*/Ta_3_N_5_ (<600 nm)	300 W Xe lamp (λ > 420 nm)	DI water adjusted by H_2_SO_4_ (pH = 3.9; 250 mL)	H_2_: 23 µmol h^−1^; O_2_: 12 µmol h^−1^	103
4	g‐C_3_N_4_ (<460 nm)	WO_3_ (<460 nm)	300 W Xe lamp	10 vol% TEOA	AQY: 0.9% (405 nm)	104
5	Rutile TiO_2_ (<406 nm)	Anatase TiO_2_ (<396 nm)	350 W Xe lamp	20 vol% methanol (80 mL)	H_2_: 324 µmol h^−1^	93
6	g‐C_3_N_4_ (<460 nm)	C,N‐TiO_2_ (<460 nm)	300 W Xe lamp (λ > 400 nm)	10 vol% TEOA (100 mL)	H_2_: 3.918 µmol h^−1^	105
7	g‐C_3_N_4_ (<460 nm)	CoTiO_3_ (<610 nm)	300 W Xe lamp (λ > 420 nm)	10 vol% ethanol	H_2_: 17.16 µmol h^−1^	106
8	Pt/g‐C_3_N_4_ (<460 nm)	WO_3_ (<460 nm)	300 W Xe lamp	10 vol% TEOA (80 mL)	H_2_: 156 µmol h^−1^	107
9	Pt/g‐C_3_N_4_ (<460 nm)	LaFeO_3_ (<598 nm)	125 W Hg lamp (λ > 420 nm)	10 vol% methanol (20 mL)	H_2_: 23.04 µmol h^−1^	108
10	Pt/g‐C_3_N_4_ (<460 nm)	W_18_O_49_ (<477 nm)	300 W Xe lamp (λ > 420 nm)	10 vol% TEOA (120 mL)	H_2_: 429.85 µmol h^−1^; AQY: 39.1% (420 nm)	100
11	Zn_0.2_Cd_0.8_S (<492 nm)	ZnO_1−_ *_x_* (<405 nm)	300 W Xe lamp (λ > 420 nm)	0.1 m Na_2_S/0.1 m Na_2_SO_3_ (100 mL)	H_2_: 2.518 mmol h^−1^	109
12	Pt/g‐C_3_N_4_ (<460 nm)	Fe_2_O_3_ (<600 nm)	350 W Xe lamp (λ > 420 nm)	15 vol% TEOA (80 mL)	H_2_: 19.9 µmol h^−1^	101
13	Zn_0.67_Cd_0.33_S (<450 nm)	ZnO (<387 nm)	300 W Xe lamp	0.1 m Na_2_S/0.1 m Na_2_SO_3_ (100 mL)	H_2_: 973 µmol h^−1^	110
14	Black P (<977 nm)	Red P (<680 nm)	20 × 10 W LED	Pure water (6 mL)	H_2_: 0.66 µmol h^−1^	111
15	Pt/Bi_2_S_3_ (<800 nm)	Bi_2_O_2.33_ (<440 nm)	500 W Xe lamp	0.1 m Na_2_S/0.1 m Na_2_SO_3_ (100 mL)	H_2_: 62.61 µmol h^−1^	112

As previously mentioned, each Z‐scheme system has its own advantages and disadvantages. PS‐A/D‐PS system confers the flexibility of PS development ascribed to its isolated units, but comes at the expense of shielding effect and backward reaction. PS‐C‐PS system renders an all‐solid‐state approach for Z‐schematic water splitting; however, costly metallic and nanocarbon electron mediators are required. On the other hand, the formation of direct Z‐scheme is highly dependent on the nature of PS units which limits the flexibility in material selection. The charge transfer mechanisms, representative systems, and key issues of each Z‐scheme mode are summarized in **Table** **10**.

**Table 10:** Charge transfer mechanisms, representative systems and key issues in different Z‐scheme modes

Gen	Charge transfer mechanisms	Representative systems	Key issues
First	PS‐A/D‐PS 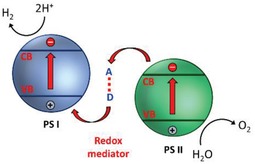	Pt/SrTiO_3_:Cr,Ta‐IO_3_−/I^−^‐Pt/WO_3_ 55 Ru/SrTiO_3_:Rh‐Fe_3_+/Fe^2+^‐WO_3_[qv: 37c] Ru/SrTiO_3_:Rh‐[Co(bpy)3]3+/2+−BiVO_4_ 54	• Strong pH dependency – IO_3_^−^/I^−^ can only function at pH more than 9 imputed to the formation of inactive I_3_^−^ at lower pH value. – Fe^3+^/Fe^2+^ is only stable at pH lower than 2.5 due to the precipitation of Fe(OH)_3_ at higher pH value. Shielding effect ascribed to the light absorption by redox mediator, i.e., aqueous Fe^3+^ exhibits strong light absorption up to 450 nm. Reverse reaction due to back donation of charge carriers. PS I and II with suitable reduction and oxidation abilities are required to drive redox reaction of different A/D pairs. Only applicable in liquid form due to the ionic nature of redox pairs which impede the potential of scalability.
Second	PS‐C‐PS 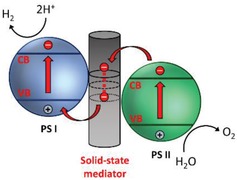	Pt/CdS‐Au‐TiO_2_ 23 Ru/SrTiO_3_:Rh‐RGO‐BiVO_4_ 25 CdS‐CQDs‐BiVO_4_ 85	Unlike the flexibility in the design of PS‐A/D‐PS system, there are constraints in the development of PS‐C‐PS system as the PS and electron mediator have to be assembled in a single unit. Costly electron mediator, for instance, metals and nanocarbons are required to dictate vectorial electron transfer. Suitable fabrication methods are essential for the development of various PS‐C‐PS systems. – To ensure intimate contact interface between PS and electron mediator. – To balance between the degree of reduction in nanocarbon mediator and the hydrophilicity of the whole composites.
Third	PS‐PS or direct Z‐scheme 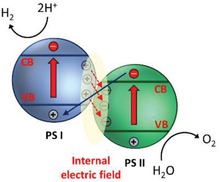	Ru/SrTiO_3_:Rh‐BiVO_4_ 27 Pt/CdS‐ZnO[qv: 26a] Pt/g‐C_3_N_4_‐W_18_O_49_ 100	Less efficient vectorial electron transfer in PS‐PS system as compared to PS‐C‐PS system due to the absence of conductor as electron mediator. Formation of internal electric field is highly dependent on the nature of PS I and PS II, i.e., PS I with higher Fermi level than PS II. Various direct Z‐scheme verification testing are required to distinguish from heterojunction typed photocatalytic systems.

## 4 Z‐Scheme Photocatalyst Sheets: Scaling Up of Leaf‐To‐Tree

### 4.1 Large‐Scale Attempts for Solar Water Splitting

**Figure 42:**
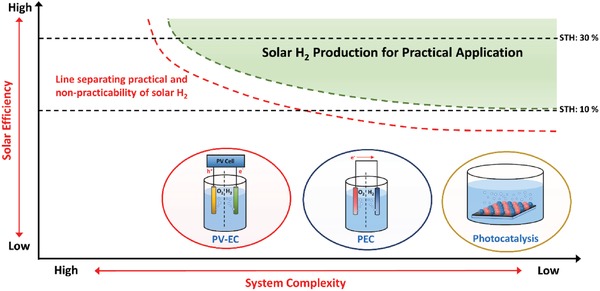
Technological map of three different solar H_2_ production approaches for practical solar energy conversion. Adapted and reproduced with permission.^3^ Copyright 2019, Royal Society of Chemistry.

**Figure 43:**
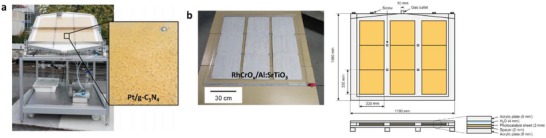
a) Large‐scale photocatalytic H_2_ production using reactor containing immobilized Pt/g‐C_3_N_4_. Adapted with permission.^113^ Copyright 2015, Wiley. b) Photocatalytic water splitting panel with immobilized RhCrO*_x_*/Al:SrTiO_3_. Adapted with permission.^9^ Copyright 2018, Elsevier.

With the progressive research efforts in developing efficient photocatalytic system for durable solar water splitting, it is timely to deliberate on scaling up of this technology for practical application. As delineated in **Figure** **42**, it can be clearly visualized that both PV‐EC and PEC confer higher complexity than photocatalysis system which indicates higher cost and higher solar efficiency target for practical application. In this aspect, solar conversion efficiency target for photocatalytic system is benchmarked at STH of 10%. Schröder et al. demonstrated the first large‐scale solar H_2_ production by immobilizing Pt‐loaded mesoporous g‐C_3_N_4_ (Pt/g‐C_3_N_4_) onto stainless steel plates using Nafion as the polymeric binder.^113^ As shown in **Figure** **43**a, a large‐scale reactor with active area of ≈0.756 m^2^ was setup by Schröder et al. for H_2_ production under natural sunlight. With the usage of plexiglass plate, the reactor allows UV transmission with wavelength above 300 nm. This designed reactor produced a total of 18.2 L of H_2_ within 30 days with measured STH of 0.12%.^113^ Nevertheless, the demonstration of feasibility in large‐scale H_2_ production using photocatalyst opens a new research interest to scale‐up this technology.

In 2018, Goto et al. reported the concept of readily extensible water splitting panel with immobilized RhCrO*_x_*/Al:SrTiO_3_ for large‐scale evolution of H_2_ gas.^9^ Figure 43b shows the flat panel reactor with 9 photocatalyst plates (33 cm^2^ each) that are arranged in 3 × 3 configuration. In the study by Goto et al., the importance of continuous gas evolution from the surface of photocatalyst sheets is highlighted, which is associated to the depth of water layer. The proposed 1 mm deep layer of water above the photocatalyst sheets allows the uninterrupted release of gas without the need of forced convection. On top of that, the water splitting panel was tilted at 10°–20° to warrant efficient release of gas and at the same time to ensure effective acceptance of sunlight. As a result, this water splitting panel demonstrated pure water splitting under natural sunlight irradiation with measured STH of 0.4%.

### 4.2 Photocatalyst Sheets with Au Conductor

**Figure 44:**
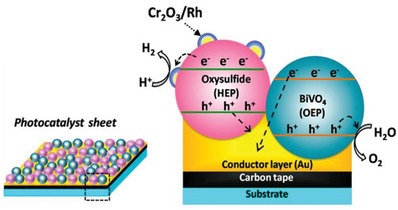
Particulate Z‐scheme photocatalyst sheets. Band configuration and plausible charge transfer mechanism in particulate Z‐scheme photocatalyst sheets. Adapted with permission.^114^ Copyright 2018, American Chemical Society.

**Figure 45:**
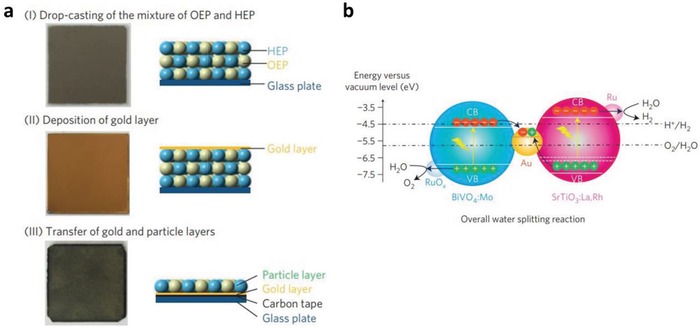
a) Preparation of Z‐scheme photocatalyst sheets using particle transfer method. b) Schematic illustration of charge transfer mechanism and overall water splitting reaction of SrTiO_3_:La,Rh/Au/BiVO_4_:Mo Z‐scheme sheets. Adapted with permission.^28^ Copyright 2016, Nature Publishing Group.

Despite numerous efforts have been attempted for scaling up of particulate photocatalyst, the efficiency of the solar H_2_ production is still low which mainly imputed to the limitation of single‐component photocatalytic configuration. On the other hand, Z‐scheme photocatalytic system which mimics the natural photosynthesis process of green plant has undoubtedly paved a giant leap toward solar H_2_ harvesting via two‐step photoexcitation configuration. As a matter of fact, these photocatalysts are mostly available in the form of powder suspension, which greatly constraints the viability of large‐scale application. As solar energy augmentation becomes a global ambition for renewable energy revolution, a serious devotion to construct Z‐scheme system with scalability is imperative. More recently, Wang et al. demonstrated the concept of particulate Z‐scheme photocatalyst sheets by constructing a two‐layer structured film: 1) top layer of photocatalysts (PS I and II) and 2) bottom layer of conductor (mediator).^29^ This ingenious arrangement of two‐layered configuration allows the top part of photocatalysts to be accessible to light irradiation and confers vectorial electron transfer via the underlying electron mediator, as shown in **Figure** **44**. A recreation of this system can be witnessed in the study by Wang et al., in which SrTiO_3_:La,Rh (PS I/HEP) and BiVO_4_:Mo (PS II/OEP) were rationally assembled on top of Au conductor to form a two‐layered photocatalyst sheets using particle transfer method (**Figure** **45**a).^28^ In comparison to powder suspension, the configuration of photocatalyst sheets can reduce the effect of H^+^/OH^−^ concentration overpotentials and pH gradient which enhance the photocatalytic performance. The thickness of photocatalysts layer should be in the optimum condition, i.e., as thin as possible to alleviate the increase in electrical resistance associated to the increase in number of grain boundaries and to be thick enough to maximize the coverage of the underneath mediator. In the work by Wang et al., particle transfer method was employed to assemble a thin layer of photocatalysts on top of a gold film. The electron shuttling ability of Z‐scheme photocatalyst sheets was further enhanced via post calcination, thus creating an intimate contact interface between the photocatalysts layer and the gold mediator. In addition, core–shell Ru/Cr_2_O_3_ co‐catalysts were loaded to promote forward photocatalytic water splitting with the suppression of oxygen reduction reaction (ORR). Consequently, Ru/Cr_2_O_3_/SrTiO_3_:La,Rh‐Au‐BiVO_4_:Mo photocatalyst sheets demonstrated STH efficiency of 1.1% with background pressure of 10 kPa, which is almost one‐order magnitude higher than ever reported Z‐scheme systems, and it serves as a benchmark for photocatalytic water splitting research.^28^

### 4.3 Photocatalyst Sheets with Nanocarbon Conductor

**Figure 46:**
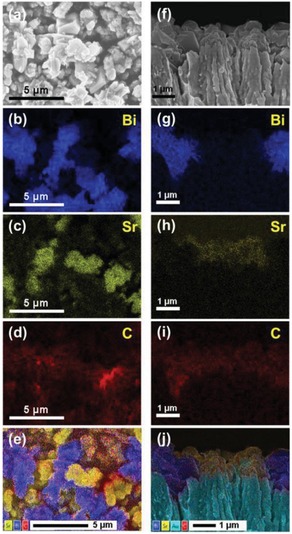
SEM–EDX mapping of a–e) top view and f–j) cross‐sectional view of SrTiO_3_:La,Rh‐C‐BiVO_4_:Mo photocatalyst sheets. Adapted with permission.^115^ Copyright 2017, American Chemical Society.

**Figure 47:**
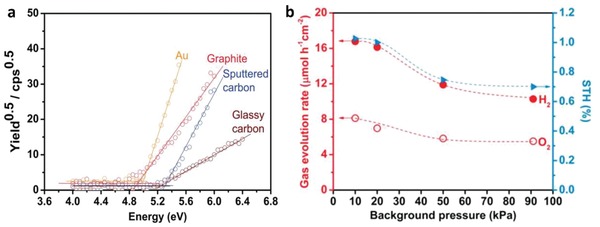
a) PESA results for Au, graphite, sputtered carbon and glassy carbon. b) Effect of background pressure on overall water splitting performance of Ru‐loaded SrTiO_3_:La,Rh‐C‐BiVO_4_:Mo photocatalyst sheets. Adapted with permission.^115^ Copyright 2017, American Chemical Society.

Similar to Au, nanocarbon is another intriguing option to serve as an electron mediator. The employment of carbon conducting layer was firstly implemented on particulate SrTiO_3_:La,Rh‐C‐BiVO_4_:Mo photocatalyst sheets.^115^ As depicted in **Figure** **46**, ≈70% of the underlying carbon base was covered by uniform distribution of Bi and Sr elements. In opposed to the expensive and rare noble metals that induce reverse reaction, carbon conductive layer provides a more promising route to relay electron in Z‐scheme sheets with its abundancy and relatively inert for backward process. As shown in the photoelectron spectroscopy in air (PESA) results of **Figure** **47**a, sputtered carbon renders a relatively high work function (5.2 eV) that is comparable to glassy carbon and higher than that of Au and graphite. This suggests the aptness of carbon conductive film in shuttling electron, which resulted in excellent overall water splitting performance from SrTiO_3_:La,Rh‐C‐BiVO_4_:Mo photocatalyst sheets.^115^ The STH value of this system was measured to be 1.0%, which is the highest among all the Z‐scheme systems operated at ambient condition. According to Figure 47b, the photocatalytic performance of carbon‐mediated particulate sheets have low dependency on background pressure, which consolidates the feasibility of this system to be operated under ambient condition.

### 4.4 Photocatalyst Sheets with Direct Z‐Scheme Configuration

**Figure 48:**
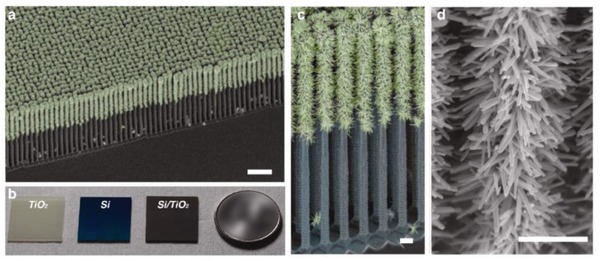
Photocatalyst sheets with direct Z‐scheme configuration. a) False‐colored SEM image of Si/TiO_2_ nanotree arrays. b) Photographs of TiO_2_, Si and Si/TiO_2_ films compared to a US quarter (right). c,d) Enlarged SEM images of Si/TiO_2_ nanotree arrays. Adapted with permission.^116^ Copyright 2013, American Chemical Society.

**Figure 49:**
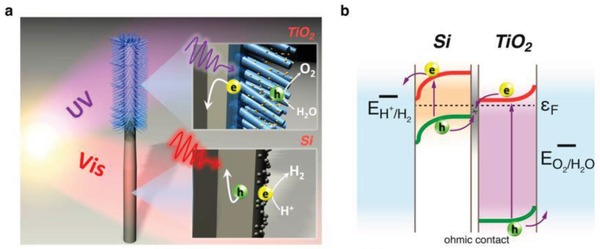
Mechanism of electron flows in Si/TiO_2_ nanotree arrays. a) Schematic illustration of charge transfer in Si/TiO_2_ nanotree arrays. b) Electronic configuration of Si/TiO_2_ nanotree arrays in overall water splitting. Adapted with permission.^116^ Copyright 2013, American Chemical Society.

Apart from Z‐scheme photocatalyst sheets that utilize conductor as electron mediator, a scale‐up version of direct Z‐scheme can be witnessed on Si/TiO_2_ film.^116^ As depicted in **Figure** **48**, it can be clearly observed that Si/TiO_2_ resembles a tree‐like structure in nanoscale. Si nanowires bear a resemblance to a trunk while TiO_2_ nanowires are the branches. Coupling between semiconductors with PS I of higher Fermi level will lead to the formation of direct Z‐scheme. In this case, internal electric field is generated at the interface of Si and TiO_2_ due to band bending, which works as an electron‐transporting channel via Ohmic contact as shown in **Figure** **49**. The unique morphology of Si/TiO_2_ bestows the advantages of large surface area and shorter charge diffusion pathway. Besides, the nanotree architecture also endows strong light harvesting ability to the system. Consequently, Si/TiO_2_ was loaded with co‐catalysts and realized overall water splitting with stoichiometric H_2_ and O_2_ evolution. In this case, Pt was loaded as the co‐catalyst for HER (Pt/Si) while IrO_2_ was employed to promote OER (IrO_2_/TiO_2_). STH efficiency of this system was measured to be 0.12% under simulated solar light.^116^ Additionally, ion‐conductive membrane can be applied in between silicon and TiO_2_ nanowires to accomplish on‐site H_2_ and O_2_ separation. In whole, this finding suggests the concept of direct Z‐scheme in macroscopic scale, which postulates a pathway for augmentation of photocatalytic water splitting other than Z‐scheme photocatalyst sheets with conductor.

Particulate Z‐scheme photocatalyst sheets have posed as a viable option for solar water splitting augmentation attributed to the ingenious arrangement of PS I/PS II layers on top of conductive film in macroscale. In stark contrast to powder suspension, water splitting reaction with photocatalyst sheets render the alleviation on the effect of H^+^ and OH^−^ concentration overpotential and pH gradient. So far, Au and carbon film have been employed as the conductive layer to govern Z‐schematic water splitting using particulate sheets. Besides, the enhancement of electron relaying between semiconductors and mediator can be realized using post calcination and surface modification, i.e., loading of co‐catalysts with protective shell. This arouses new interest in the development of this new generation Z‐scheme system attributed to its scalability and commercial potential. A summary of Z‐scheme photocatalyst sheets for overall water splitting is tabulated in **Table** **11**.

**Table 11:** Summary of Z‐scheme photocatalyst sheets for overall water splitting

Entry	PS I (available λ)	PS II (available λ)	M	Light source	Reaction condition	Efficiency	Ref.
1	Ru/SrTiO_3_:Rh,La (<500 nm)	RuO*_x_*/BiVO_4_ (<520 nm)	Au	300 W Xe lamp (λ > 420 nm)	DI water adjusted by H_2_SO_4_ (pH = 3.5; 40 mL)	H_2_: 4.5 µmol h^−1^ cm^−2^; O_2_: 2.2 µmol h^−1^ cm^−2^; AQY: 5.9% (418 nm); STH: 0.2%	29
2	Ru/Cr_2_O_3_/SrTiO_3_:Rh,La (<500 nm)	RuO*_x_*/BiVO_4_:Mo (<510 nm)	Au	300 W Xe lamp (λ > 420 nm)	DI water adjusted by H_2_SO_4_ (pH = 3.5; 40 mL) Pressure: 5 kPa	AQY: 33% (419 nm); STH: 1.1%	28
3	RhCrO*_x_*/ LaMg_1/3_Ta_2/3_O_2_N (<600 nm)	BiVO_4_:Mo (<510 nm)	Au	300 W Xe lamp (λ > 420 nm)	DI water (40 mL)	STH: 1 × 10^−3^%	117
4	RhCr_2_O_3_/La_5_Ti_2_CuS_5_O_7_ (<660 nm)	BiVO_4_ (<520 nm)	Au	300 W Xe lamp (λ > 420 nm)	DI water (pH = 6.9; 40 mL)	AQY: 4.9% (420 nm); STH: 0.11%	114
5	Ru/Cr_2_O_3_/SrTiO_3_:Rh,La (<500 nm)	RuO*_x_*/BiVO_4_:Mo (<510 nm)	C	300 W Xe lamp (λ > 420 nm)	DI water adjusted by H_2_SO_4_ (pH = 3.5; 40 mL) Pressure: 91 kPa	STH: 1.0%	115
6	Pt/Si (<1060 nm)	IrO_2_/TiO_2_ (<387 nm)	–	300 W Xe lamp	0.5 m H_2_SO_4_	H_2_: 2.1 µmol h^−1^; O_2_: 1.1 µmol h^−1^	116

Despite the potential of Z‐scheme photocatalyt sheets in realizing large‐scale solar water splitting, numerous challenges are faced for instance: 1) improved photocatalytic efficiency of materials, 2) enhanced interfacial contact between photocatalysts layer and electron mediator, 3) reduced internal resistance loss due to increase in grain boundaries and 4) minimized overall efficiency loss when scaling up. All the requirements are essential for reducing the cost of solar H_2_ production to be commercially feasible. Strategic approach to improve the photocatalytic performance of Z‐scheme system will be outlined henceforth in Section 5. However, it is rather difficult to construct particulate photocatalyst sheets with intimate contact interface between photocatalysts and conductor layer. Post calcination with suitable temperature is essential to enhance the contact interface between the two layers while maintaining their intrinsic properties. The thickness of photocatalysts layer should be as thin as possible so that the electrical resistance associated to the increase in number of grain boundaries can be avoided. At the same time, photocatalysts layer must be dense enough to cover the underlying conductor layer. On top of that, special consideration has to be focused on the construction of scaled up device. Overall efficiency loss associated with the increase in Ohmic resistance and formation of spatial defects is inevitable during the scaling up process.^3^ Thus, future direction in the development of particulate Z‐scheme photocatalyst sheets should focus on addressing these challenges.

## 5 Strategic Approach to Improve Z‐Schematic Water Splitting

**Figure 50:**
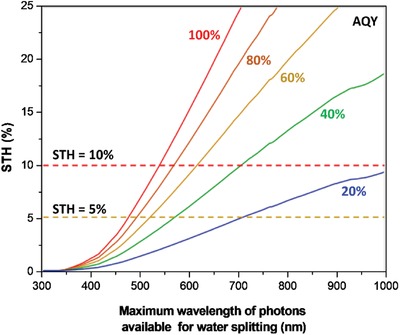
Relationship between STH conversion efficiency and maximum wavelength of photons available for water splitting at different values of AQY for photocatalytic water splitting. Adapted and reproduced with permission.^119^ Copyright 2014, Springer.

As aforementioned in the previous sections, Z‐scheme photocatalytic system confers unique band configuration with cascade electron transport profile which demonstrated water splitting advancement as compared to single‐component and heterojunction‐typed photocatalysts. Even so, the efficiency of current Z‐scheme systems is still far from being economically feasible for commercialization. According to techno‐economical evaluation of solar H_2_ produced from PEC and photocatalysis systems by the United States Department of Energy, a STH value of 10% with 10 years of lifetime is required to meet the target H_2_ price of 1.6 USD kg^−1^.^118^ As shown in **Figure** **50**, it can be clearly visualized that photocatalyst requires minimum absorption wavelength of 600 nm with AQY of 60% in order to achieve STH of 10%. In other words, photocatalyst with wavelength shorter than 600 nm will need even higher value of AQY to realize this goal.

**Figure 51:**
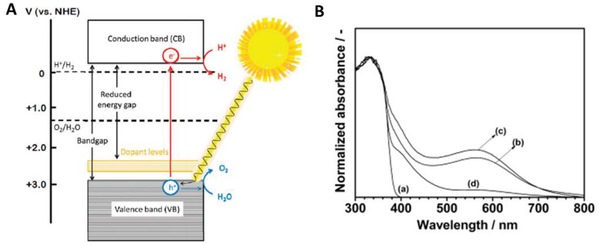
A) Electronic band structure of photocatalysts after interstitial doping of heteroatoms. Adapted with permission.^122^ Copyright 2012, American Chemical Society. B) UV–vis DRS of a) SrTiO_3_, b) SrTiO_3_:Rh, c,d) SrTiO_3_:La/Rh prepared by one‐step SSR and two‐step SSR. Adapted with permission.^71^ Copyright 2014, American Chemical Society.

The interstitial doping of transition metals with partially filled d orbitals as foreign heteroatoms into d^0^‐based or d^10^‐based oxides is one of the many research efforts in bandgap reduction of materials.^120^ In this context, the hybridization of d orbitals from transition metal cation can lead to the formation of a new energy state that is allocated above the O 2p energy level. In other words, the bandgap of the semiconductor is narrowed ascribed to the shallower VB and the light absorption capability is redshifted (**Figure** **51**a). A typical example of this approach can be witnessed in an all‐solid‐state Z‐scheme system reported by Wang et al., in which Rh‐ and La‐codoped SrTiO_3_ is used as HEP.^71^ As shown in Figure 51B, the light absorption of Rh‐doped SrTiO_3_ is redshifted as compared to pristine SrTiO_3_ attributed to transition of photogenerated electrons from the hybridized state of Rh^3+^ to the CB which mainly constituted of Ti 3d orbital. However, the presence of Rh^4+^ species is detrimental due to its inactivity for H_2_ production and serves as charge recombination center for the photogenerated carriers. Meanwhile, the introduction of codoping of La atoms can suppress the formation of oxygen defects due to the partial substitution of Ti^4+^ sites. In 2016, Kobayashi reported an Ag‐mediated Z‐scheme system that can be operated up to 740 nm.^121^ The incorporation of ZnRh_2_O_4_ (*E*_g_: 1.2 eV) and Aurivilluis‐typed Bi_4_V_2_O_11_ as small bandgap HEP and OEP can harness nearly the whole visible light spectrum for overall water splitting. Hence, this result offers a new approach for the devise of Z‐scheme system to be operated under higher wavelength of the solar spectrum.

**Figure 52:**
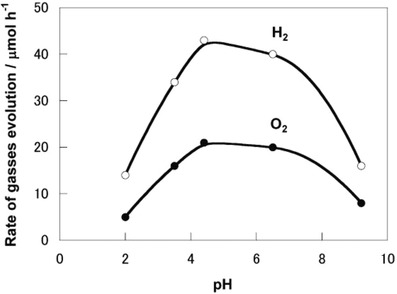
Photocatalytic overall water splitting of WO_3_‐based Z‐scheme redox system. Rate of gas evolution over PtO*_x_*/H‐Cs‐WO_3_ as OEP and Pt/SrTiO_3_:Cr,Ta as HEP in I_3_^−^/I^−^ and IO_3_^−^/I^−^ system under different pH. Adapted with permission.[qv: 36c] Copyright 2013, Royal Society of Chemistry.

As is known, WO_3_ has been widely documented as an efficient OEP. Sayama et al. firstly implemented Fe^3+^/Fe^2+^ ionic Z‐scheme system by using RuO_2_‐WO_3_ as OEP.^123^ It has been stated in the previous discussion that the presence of inactive I_3_^−^ in IO_3_^−^/I^−^ redox cycle will significantly suppress the reduction process from OEP. One of the prominent ways is to control the pH of solution to be more than 9 which in turn hinders the production of I_3_^−^. However, this will greatly limit the application of IO_3_^−^/I^−^ redox system in a wide pH range. In an effort to overcome this problem, surface treatment of WO_3_ by Cs^+^ salt and H^+^ exchange reaction yielded the formation of H‐Cs‐WO_3_ with ion‐exchangeable sites.[qv: 36c] Remarkably, the presence of these ion‐exchangeable sites can promote the reduction of IO_3_^−^ and at the same time enhance the water oxidation performance of WO_3_. The photocatalytic O_2_ evolution of H‐Cs‐WO_3_ in IO_3_^−^ solution resulted in AQY of 20% under monochromatic 420 nm irradiation. Besides, PtO*_x_*‐loaded H‐Cs‐WO_3_ exhibited high O_2_ evolution in solution containing I_3_^−^ ions in stark contrast to the negligible activity from untreated PtO*_x_*/WO_3_. It can be speculated that the presence of ion‐exchangeable sites induced by the surface treatment can promote the reduction of I_3_^−^ ions as well. Consequently, overall water splitting with stoichiometric evolution of H_2_ and O_2_ was demonstrated using NaI solution containing PtO*_x_*/H‐Cs‐WO_3_ as OEP and Pt/SrTiO_3_:Cr,Ta as HEP over a wider pH range (**Figure** **52**). Additionally, surface treated Fe‐H‐Cs‐WO_3_ works well under VO_2_^+^/VO^2+^ redox system, resulting in STH of 0.06% under AM 1.5 simulated solar light irradiation.[qv: 37b]

**Figure 53:**
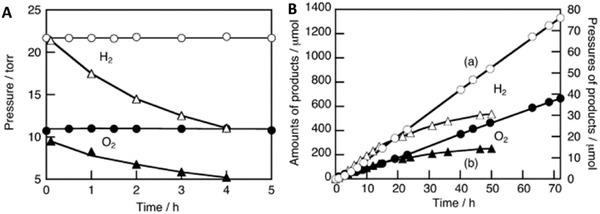
A) Consumptions of H_2_ and O_2_ due to backward reactions on Pt/SrTiO_3_:Rh‐BiVO_4_ (Triangle) and Ru/SrTiO_3_:Rh‐BiVO_4_ (Circle). B) Overall water splitting of a) Ru/SrTiO_3_:Rh‐BiVO_4_ and b) Pt/SrTiO_3_:Rh‐BiVO_4_. Adapted with permission.[qv: 37c] Copyright 2008, Elsevier.

The loading of co‐catalysts is indispensable to promote photocatalytic overall water splitting associated to the decrease in kinetic barrier and facilitate forward reaction. Besides, the presence of uniformly dispersed co‐catalysts in sub‐nanoscale can provide surface active sites and maximize the atom‐utilization efficiency ascribed to the minimum agglomeration of nanoparticles.^124^ In general, co‐catalysts that are suitable for HER include Pt, Ru, Ni, Rh and Ir. On the other hand, oxides form of Ru, Ni, Ir, Co and Fe can propel OER. In the earlier studies of redox‐mediated Z‐scheme systems, Pt is often employed as co‐catalyst for enhancing HER process.^35^, ^55^, ^56^ The low overpotential of Pt can efficiently catalyze forward HER reaction and has been widely incorporated in many HEPs for photocatalytic H_2_ half reaction. However, Pt is also a strong co‐catalyst for driving backward reaction of overall water splitting, i.e., the ORR process or water formation. In a study on the effect of Pt on Fe^3+^/Fe^2+^ mediated Z‐scheme system by Sasaki et al., it was reported that Pt exhibited severe backward reaction that hindered the efficiency of overall water splitting (**Figure** **53**). Even though it was found out that the presence of Fe^2+^ in Z‐scheme redox system can suppress the backward reaction of Pt/SrTiO_3_:Rh‐BiVO_4_ due to the adsorption of [Fe(OH)(H_2_O)_5_]^2+^, the water oxidation performance of the system is greatly reduced.^16^ In contrast, Sasaki et al. demonstrated that Ru co‐catalyst is more efficient as compared to Pt in term of photocatalytic overall water splitting, as delineated in Figure 53. The effect of backward reaction ascribed to ORR when employing Ru as co‐catalysts is less significant, making Ru the better candidates to drive overall water splitting.

**Figure 54:**
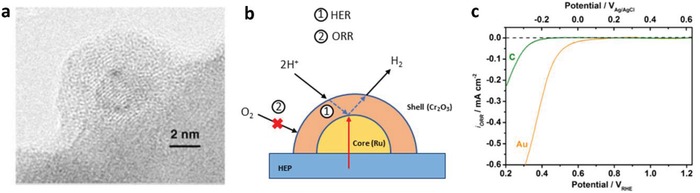
a) HRTEM image of Rh/Cr_2_O_3_‐loaded (Ga_1−_ *_x_*Zn*_x_*)(N_1−_ *_x_*O*_x_*). b) Schematic of overall water splitting mechanism on nanostructured core/shell co‐catalyst loaded HEP. Adapted and reproduced with permission.^125^ Copyright 2006, Wiley. c) ORR activity of C/Ti and Au/Ti electrodes. Adapted with permission.^115^ Copyright 2017, American Chemical Society.

In an effort to suppress the undesirable backward reaction from ORR and redox couples, Cr species is introduced to form nanostructured core/shell metal/Cr_2_O_3_ co‐catalyst to promote forward reaction. The early work of Maeda et al. in utilizing Rh/Cr_2_O_3_ as co‐catalyst on (Ga_1−_ *_x_*Zn*_x_*)(N_1−_ *_x_*O*_x_*) solid solution effectively suppressed ORR and achieved overall water splitting.^125^ As shown in **Figure** **54**a, Cr species forms a nanosized shell that covers the metal core. Owing to the properties of the Cr_2_O_3_ as protective shell, water can be diffused into the metal core and the H_2_ product is freely to be released. At the same time, the protective shell prohibits O_2_ produced from water oxidation to permeate through the layer, which in turn hinders the tendency of ORR process. In a recent work by Wang et al., carbon conductive film was incorporated as electron mediator to interface SrTiO_3_:La,Rh, and BiVO_4_:Mo as Z‐scheme photocatalyst sheet.^115^ The employment of carbon film can minimize the tendency of reverse reaction as compared to metallic conductor such as Au and Rh. Even though metallic conductors possess large work function to be served as electron mediator, the reverse reaction imposed by metal under escalated background pressure is severe (Figure 54c). On top of that, Ru/Cr_2_O_3_ was chosen as the nanostructured co‐catalyst to prevent ORR effect. As a result, the particulate Z‐scheme photocatalyst sheets demonstrated a high STH value of 1.0% under ambient pressure.^115^ Another typical example of Cr species in inhibiting backward reaction can be witnessed in a redox IO_3_^−^/I^−^ Z‐scheme system with Ta_3_N_5_ as HEP and TaON as OEP.^126^ The loading of Cr_2_O_3_ shell onto Pt metal can serve as an efficient co‐catalyst in driving overall water splitting with negligible competitive IO_3_^−^ reduction. This technology presents a viable approach to incorporate co‐catalyst that is suitable to drive overall water splitting in Z‐scheme system.

## 6 Overall Conclusion and Outlook

In conclusion, photocatalysts with broad light absorption range, efficient charge separation, and strong redox ability are essential to govern an efficient photocatalytic water splitting reaction. However, it is rather difficult for a single‐component photocatalytic system to simultaneously exhibit all these properties. Z‐scheme photocatalytic system renders a viable approach to overcome these problems by allowing vectorial electron transfer attributed to the unique electronic configuration. As a result, electrons and holes will be accommodated at the highest possible CB and lowest VB, which provide strong reduction and oxidation abilities. Hitherto, Z‐scheme systems can be generally divided into three generations: 1) PS‐A/D‐PS, 2) PS‐C‐PS, and 3) PS‐PS or direct Z‐scheme system. The pioneering work of Z‐scheme photocatalytic water splitting focuses on utilizing redox mediator for cascade electron flow, which is also known as the PS‐A/D‐PS (first generation) system. The nature of ionic pairs in liquid form allows PS I and PS II to be constructed independently in PS‐A/D‐PS system. Thus, the configuration of PS I and PS II can be freely modified and modulated as two isolated units. This feature of PS‐A/D‐PS system enables high degree of freedom in fabrication of photosystems. However, the strong pH dependency, shielding effect, and reverse reaction associated to redox pairs greatly limit the application of PS‐A/D‐PS system. With regard to this problem, surface modification of photocatalysts has been introduced to minimize the effect of reverse process imputed to the reversibility of ionic pairs.

Recently, the employment of all‐solid‐state Z‐scheme system becomes more prominent due to the suppression of backward reaction and shielding effect with the absence of redox pairs. PS‐C‐PS and PS‐PS Z‐scheme are the two commonly studied systems in photocatalytic water splitting. In view of PS‐C‐PS (second‐generation) system, an external conductor is required for the electron relaying. The competency of metal and nanocarbon materials in driving electron transfer has made them the ideal candidate as a conductor. The path for electron transfer is also shortened for all‐solid‐state Z‐scheme system ascribed to the electron flow via low resistance contact which is the Ohmic contact. For years, PS‐C‐PS photocatalytic water splitting system has been widely conducted by employing metals as the electron mediator. As of recent, carbonaceous materials such as graphene and CNTs are relative new addition to the family of electron mediator for Z‐scheme. Constructing PS‐C‐PS system with conductive carbon as mediator, albeit still not as prevalence as the conventional metallic conductor, have increased exponentially attributed to the competency of carbonaceous materials in shuttling cascade electron flow. However, attention should be given on the fabrication of conductive carbon‐driven Z‐scheme to avoid formation of defects on the graphitic structure during the synthesis process.

For the PS‐PS (third generation) or direct Z‐scheme system, the formation of internal electric field is highly dependent on the nature of semiconductors. Coupling of two semiconductors with PS I of higher Fermi level will induce negatively charged interface, which eventually leads to the formation of direct Z‐scheme. Conversely, when PS I has a lower Fermi level, a type‐II heterojunction will be formed. The quasi‐continuous energy state at the solid–solid interface of PS‐PS system can bestow internal electric field for vectorial electron flow with low resistance. In addition, verification testing such as metal loading, radical trapping experiment, and XPS methods are important to distinguish the presence of direct Z‐scheme configuration rather than a conventional type‐II heterojunction. Since the turn of a new decade, attention has been drawn into the augmentation of technologies. Particulate Z‐scheme photocatalyst sheets are the new promising system with potential scalability in overall water splitting. Even so, there is still much vagueness in the charge transfer dynamics of Z‐scheme system. Besides, mediator should be examined on not only their electrical performance but also their work function with the coupled semiconductors. In the hopeful future, highly efficient Z‐scheme system can be methodically realized in driving large‐scale photocatalytic water splitting for H_2_ generation.

## Conflict of Interest

The authors declare no conflict of interest.
